# Formulation approaches for colon-specific drug delivery: conventional to nanocarrier systems

**DOI:** 10.1039/d5ra05194k

**Published:** 2026-02-23

**Authors:** Ashish Sriram Mishra, Bhavna Ghosh, Sivakumar Ponnurengam Malliappan, Gouranga Dutta, Manimaran Vasanthan

**Affiliations:** a Department of Pharmaceutical Quality Assurance, SRM College of Pharmacy, SRM Institute of Science and Technology Kattankulathur Chengalpattu 603203 Tamil Nadu India manimarv@srmist.edu.in; b Department of Pharmaceutical Analysis, School of Pharmaceutical Sciences, Siksha ‘O’ Anusandhan Bhubaneswar Odisha India; c School of Medicine and Pharmacy, Institute of Research and Development, Duy Tan University Da Nang Vietnam; d Department of Pharmaceutics, Bharat Technology Uluberia Howrah India

## Abstract

The development of colon-targeted drug delivery systems (CDDS) has gained increasing attention due to their potential to improve therapeutic outcomes for diseases such as inflammatory bowel disease (IBD), irritable bowel syndrome (IBS), colorectal cancer, and other colonic disorders. Targeted delivery to the colon offers the advantages of site-specific action, reduced systemic side effects, and improved patient compliance. However, several physiological barriers, such as variable pH, microbial metabolism, enzymatic degradation, and transit time, pose significant formulation challenges. This review provides a comprehensive overview of conventional and advanced formulation strategies for colon-targeted drug delivery. Various approaches, including pH-responsive systems, time-dependent delivery, microbially triggered systems, prodrug strategies, pressure-controlled devices, and nanotechnology-based delivery platforms, are critically discussed. Additionally, the emerging role of natural polysaccharide-based systems and innovative hybrid formulations is highlighted. Comparative analysis of different strategies is presented to guide future formulation design. Advancements in nanotechnology and biomaterials offer promising opportunities to overcome existing challenges and enable precise and efficient colon-targeted drug delivery. Further research is warranted to translate these innovative systems into clinically viable therapies with improved efficacy and patient outcomes.

## Abstract 2

Colon-targeted drug delivery enables site-specific therapy for colonic disorders with reduced systemic effects. This review highlights conventional and advanced strategies, including stimulus-responsive, polysaccharide-based, and nanocarrier systems.

## Graphical abstract

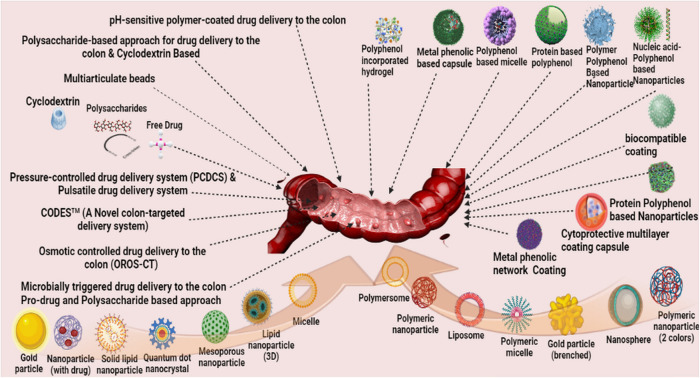

## 1 Introduction

Colon-targeted drug delivery pertains to dispensing medications specifically targeting the colon or the large intestine for localized therapeutic effect or systemic absorption. Colon-specific drug delivery holds several rationales for its significance, including the potential to optimize treatment efficacy for several colon-specific inflammatory conditions and minimize any unfavorable systemic side effects.^1,2^ The targeted delivery of medication to the colon can potentially reduce the required dosage for treatment. Several therapeutics not absorbed in the upper gastrointestinal tract (GIT) may have better absorption when introduced to the colon, where absorption is more efficient. The investigation and advancement of drug delivery systems that target the colon is a noteworthy area of study, owing to its capacity to augment the efficacy and safety of treatments for colon-specific disorders and mitigate the disease burden for individuals.^1,2^ Due to the unique characteristics and environment of the digestive tract and colon, drug delivery to the digestive tract and colon encounters several severe obstacles. Obtaining the required bioavailability in the intestinal areas while minimizing toxicity to the helping the bacteria in that region is a significant issue. Protecting the medication is a top priority for the colon-targeted drug delivery system. Hydrolysis and enzymatic breakdown of peptide medications in the duodenum and jejunum increase their systemic bioavailability and are known as colon-specific drug delivery systems (CDDS). Drugs absorbed in the colon have a prolonged half-life and are sensitive to absorption enhancers. Many techniques are available for CDDS, but oral delivery is the most effective and is often preferred.^3–5^ Medications taken through the rectum are more likely to be absorbed and removed by the colon. Therefore, accessing the proximal colon with a rectal delivery is more challenging due to unpleasant effects for some people, and a regular dose is not always preferable.^6–8^

The effectiveness of some medications is often attributed to their topical use despite being absorbed through the digestive system. Several factors influence drug concentration in the colon, *i.e.*, formulation attributes, retrograde spreading, and retention time. Colonic contents are relatively thick and poorly mixed due to the colon's high water absorption capacity. This makes it difficult for most medications to overcome the permeable barrier and reach their target.^9,10^ It is estimated that the human colon has about 400 different types of bacteria and the number of bacteria inside at 10^10^ per gram of intestinal contents. This bacterial population is thought responsible for eliminating many of the azo compounds compound slots by cleaving the glycosides process. Research evidence shows that these pathways are responsible for the metabolism of many drugs and cause unwanted health disorders.^11,12^ Preserving and treating health issues related to bacteria is the focus of research into using nanoparticles in colon-based medication delivery.^13^ Thus, this article explores the utilization of nano-formulations to deliver medications and pharmaceuticals to the colon, intending to enhance colonic microbiota and decrease the risk of gastrointestinal imbalance.

Inflammatory bowel disease (IBD) is one condition whereby localized drug delivery to the colon rather than systemic administration is preferable for optimal therapeutic success. CDDS has the potential to aid people with irritable bowel syndrome (IBS), Crohn's disease (CD), and colorectal cancer (CRC). Certain drugs may be absorbed mainly in the colon rather than the stomach or small intestine. Proteins and peptides are usually destroyed in the stomach's acidic environment and may be absorbed systemically by the colon's mucosa.^14^ The conventional delivery approaches further possess several limitations, including sensitivity to changes in pH, enzymatic activation, receptor regulators, and further propulsion by magnetism. On this note, colon-targeted delivery methods have gained much attention since they can increase the bioavailability of macromolecules *via* the oral route of administration.^15^ By protecting peptide drugs from breakdown in the small intestine and releasing them in the colon, CDDS maximizes systemic absorption. CDDS is most effective when administered orally. In addition, drugs administered rectally can reach the colon quickly but may cause discomfort and reduce patient compliance if pain is experienced.^16^ In addition, intrarectal administration enables significant intestinal absorption of drugs with systemic and topical effects using liquids, foams, or suppositories. Therefore, it is essential to specify new formulation possessions that allow for the controlled, prolonged, and targeted delivery of medication in the colon to improve their effectiveness and bioavailability.^5,17,18^

As we age, our health tends to deteriorate due to several factors. Enzymatic and biological processes slow down, leading to digestion and stomach problems that persist longer, with the digestive and absorption regions being most commonly affected. Additionally, the accumulation of toxicity from medications and chemicals in our food contributes to this deterioration.^19,20^ Drug delivery systems tailored to the colon can improve the lives of people suffering from various medical conditions. Some proteins and peptides are resistant to the stomach's high pH and may be absorbed by the intestinal mucosa.^21^ The formulation strategies may focus on the digestive process, namely the colonic bacterial population, digestive enzymes, and the stomach's acidity-to-chyme ratio. Custom formulations for the colon may improve medication dispersion, effectively treating gastrointestinal problems.^22^ Moreover, colon-specific formulations enhance the transport and duration of the impact of protein- and peptide-based medicines because hepatic metabolism strictly controls the chemical and enzymatic degradation of upper gastrointestinal tract contents. Treating severe colon disorders would be much less complicated if medications were designed specifically for the colon.^23,24^

This review discusses explicitly several formulation designs utilized for traditional and innovative CDDS methods. The main objective is to target and transport the drug to the colon, intending to evaluate its effectiveness in treating disorders that specifically affect the colon. Colon-targeted drug delivery systems offer precise medication administration, reduced systemic side effects, and improved therapeutic efficacy. Scientists are now studying novel approaches to administering medications that have improved drug release kinetics, durability, and biological compatibility. The article explores traditional methods and informs readers about innovative carrier-based drug delivery techniques targeting the colon. We compared conventional and novel methods of administering drugs to the colon and also examined the advantages of using unique carrier-based delivery systems for the colon. Combining these systems with compatible therapeutic methods can enhance the effectiveness of treatment.

## 2 Overview of physiology of colon and drug's absorption factors

The colon is the last portion of the digestive system and plays an essential role in absorbing water, electrolytes, and micronutrients from undigested food. It is separated into various sections, including the cecum, ascending colon, transverse colon, descending colon, sigmoid colon, and rectum. Transporters, ion channels, enzymes, and bacteria all play a role in the absorption and secretion functions of the organism. The colon is a desirable site for drug delivery because of its large surface area, delayed transit duration, and neutral pH environment. The colonic epithelium is distinguished from the small intestine by ion transporters and enzymes and a greater blood flow. In addition, the colon's microbiota can play an essential role in drug metabolism and absorption by producing active metabolites absorbed by the colon.^25^

In recent years, scientists have emphasized the colon, also known as the large intestine, as a site of drug absorption. Along a concentration gradient, pharmaceuticals can travel through the intestinal epithelium without requiring any energy. Active transport is the mechanism by which drugs must use energy to pass through the colonic epithelium against a concentration gradient.^26^ The P-glycoprotein (P-gp) transporter is one of the most well-known active transporters in the colon; it is responsible for the efflux of medications from the colon back into the lumen, thereby decreasing their bioavailability. Other active transporters, such as multidrug resistance-associated protein 2 (MRP2), organic anion transporter (OAT), and organic cation transporter (OCT), contribute to colonic medication absorption. A comprehensive understanding of these processes is necessary to formulate medications intended for colonic administration.

In healthy individuals, the colon chyme transit time can range from a few hours to three days. The longitudinal layer consists of three broad bands that extend from the cecum to the rectosigmoid junction (taeniae).^27^ At the rectosigmoid junction, three taeniae encompass the rectum. The corrugator cutis ani muscle creates the anal canal's longitudinal muscle layer. The taeniae coli of monkeys, horses, guinea pigs, and rabbits serve as suspension cables for the muscular arches in their bodies. A circular contraction would reduce the lumen's diameter by a third if the longitudinal muscles were concentric. The coordinated contraction of longitudinal and circular muscles causes peristalsis. The mesentery provides support for the colon. A feeble mesentery obstructs the cecum and colon. Some people have a drooping transverse colon or a feeble sigmoid colon because the mesentery of the transverse and sigmoid colons is more apparent.^28^

Moreover, concerns about the drug's stability in the intestines may arise. The effectiveness of the therapy may be diminished owing to nonspecific interactions between the drug and the contents of the colon, including meal remnants, digestive juices, mucus, and excrement. The intestinal tract may also contain bacterial enzymes that render the medicine ineffective.^29^ Several variables may influence colon drug bioavailability and research toward developing colon-specific medication delivery systems.

### 2.1 Biological aspects

About 1.5 meters in length, the human large intestine comprises three colon sections (the ascending, transverse, and descending colons) plus a short segment of the rectum. The colon's physiological makeup differs from the rest of the digestive tract. However, the colon's unpredictable transit of food and dose forms presents a challenge to developing colonic medication delivery devices.^30,31^ The average human daily diet weighs around 1.6 kg and consists mainly of indigestible proteins, carbohydrates, and fats, leading to a variable time from intestinal to colonic transit. As a result of its high water-absorption ability, the colon may absorb up to 90% of incoming water. The estimated volume range for colonic fluid is 1–44 ml, with a mean value of around 13 ml. Transfer of drugs to the colon is facilitated by time-dependent release mechanisms reliant on GI transit intervals. With a modest variance between 2 and 6 hours amongst individuals, the average intestinal transit time is 4 h.^32^

In addition, research indicates that people with colon issues, such as active UC, had much quicker intestinal transit.^32^ Standard oral formulations might have a shorter transit time in IBD patients with diarrhea and intestinal resection. This decreased transit time may minimize the exposure of diseased colon segments to topically active oral drugs; hence, the therapeutic effect against the active disease should be significantly diminished.^33^

### 2.2 Colon viscosity of luminal content

The luminal contents of the colon are denser than those of the upper GIT, which complicates the liquidation of CDDS. The solubility and mucosal absorption of drugs are reduced when they move from the ascending to the descending colon, which reduces the medicine's viscosity and, therefore, its capacity to reach and infect disease-causing bacteria in the colon.^34^

### 2.3 Colonic pH

Distinct parts of the digestive tract have varying pH levels. For instance, the stomach is highly acidic, with a pH of 1–2. At the same time, the distal small intestine can have a pH as high as 7.6 due to fatty acids produced by bacteria in the digestive tract from the conversion of polysaccharides.^35^ Medications that contain polysaccharides may have an impact on the pH level of the gut. Laxatives, such as lactulose, are fermented by bacteria in the colon, which can lead to a decrease in pH level and the production of lactic acid. Studies have also shown that intestinal diseases, such as ulcerative colitis (UC), can cause changes in the pH level of the colon.^36^

### 2.4 Metabolism of enzymes and mucosal barrier

Bacteria like *Escherichia coli* and *Clostridium* species are only a few of the over 400 species that call the colon home. These bacteria possess an abundance of metabolic enzymes, both hydrolytic and reductive. When broken down by stomach enzymes, drugs may become pharmacologically active, inactive, or dangerous metabolites.^37^ Mucus, or mucin, is the major component of the hydrogel layer comprising mucus. In humans, the thickness of the mucosal layer ranges from 10 to 200 millimeters (from the jejunum to the colon). The mucus lubricates chyme, shields the epithelium from damage, and binds and prevents pathogens from entering epithelial cells.^38^

### 2.5 Design considerations

The colon contains between 1 and 45 ml of dissolvable colon fluid; hence, the solubility and dosage of medicine are crucial determinants in determining its colonic bioavailability. The biodistribution and bioavailability of medication in the colon are influenced by the treatment's physicochemical qualities, dosage, and dosing type. Useris^®^ and Entocort EC are the authorized budesonide treatments for UC and CD.^39^

### 2.6 Physiological barriers and foundational design considerations in colon-targeted drug delivery systems

**Fig. 1:**
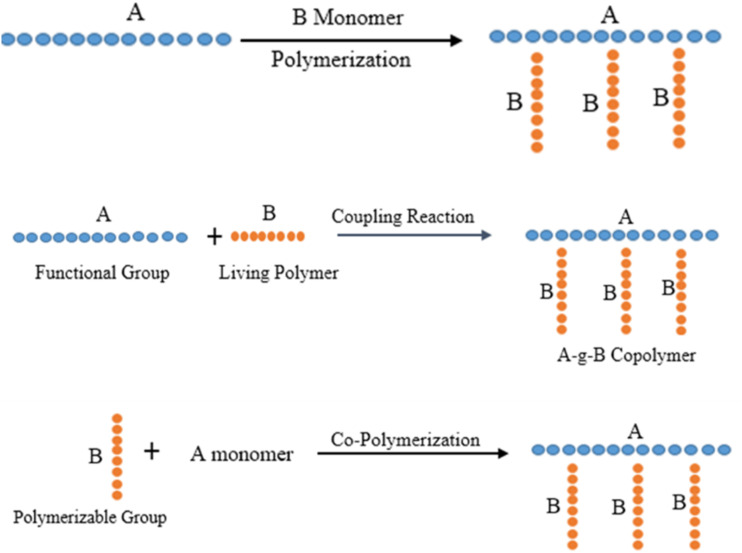
(a): ‘Grafting To’ technique-pre-polymerized chains are attached to reactive core polymers; (b): ‘Grafting From’ technique: a conducting polymer core synthesized with initiation side functions as a macro-initiator from where side chains are grown; (c):‘Grafting Through’ technique: after polymerization, macromonomers are synthesized to form core polymer.

The efficacy of colon-targeted drug delivery systems (CDDS) is governed by a complex interplay of physiological barriers that differ markedly from those influencing conventional oral drug delivery. These barriers include pH variability, microbial metabolism, enzymatic degradation, and gastrointestinal transit time, each exerting distinct and often interdependent effects on drug release and absorption.^40,41^ These foundational design considerations underpin polymer selection and formulation strategies for colon-targeted drug delivery systems, as schematically illustrated in Fig. 1. A clear differentiation and mechanistic understanding of these factors are essential for the rational design of robust CDDS capable of performing consistently under diverse physiological and pathological conditions.

Among these barriers, colonic pH variability has received significant attention due to its direct impact on pH-responsive polymers. Quantitative clinical studies indicate that colonic pH typically ranges from 6.2 to 6.8 in the proximal colon and may increase to 7.2–7.4 in distal regions under healthy conditions.^42^ However, in inflammatory bowel disease and other pathological states, colonic pH may decrease to 5.2–6.5 as a consequence of inflammation, altered microbial metabolism, and increased short-chain fatty acid production.^43^ Such variability compromises the reliability of purely pH-dependent delivery systems and explains their inconsistent performance in clinical settings.

Microbial metabolism constitutes another critical determinant of CDDS performance. The colon harbors a dense and metabolically diverse microbiota capable of producing enzymes such as azo-reductases, glycosidases, and esterases that are exploited for site-specific drug release.^44^ However, microbial composition varies significantly across individuals due to diet, age, antibiotic exposure, disease severity, and geographic factors, leading to heterogeneous enzymatic activity and variable drug release profiles.^45^ This inter-individual heterogeneity presents a major challenge for microbial-triggered systems and necessitates the development of adaptable or multi-trigger strategies.

In addition to microbial metabolism, enzymatic degradation of both drugs and carrier materials influences CDDS efficacy. Enzymatic activity in the colon differs not only between individuals but also between *in vitro* and *in vivo* environments. While fecal slurry and caecal content models provide valuable mechanistic insights, they often fail to replicate the dynamic complexity of the human colon, leading to discrepancies in degradation rates and release kinetics.^46^ These limitations underscore the need for improved translational models that more accurately predict *in vivo* behavior.

Gastrointestinal transit time represents a further source of variability affecting CDDS performance. Time-dependent delivery systems rely on predictable gastric emptying and intestinal transit; however, clinical data demonstrate that gastric emptying time can vary widely—from approximately 30 minutes to more than 6 hours—depending on food intake, circadian rhythm, stress, and disease state.^47^ Such variability undermines the predictability of time-controlled systems and contributes to premature or delayed drug release.

Pathological conditions further exacerbate transit-related challenges. Inflammatory states, diarrhea, and post-surgical resection are commonly associated with accelerated intestinal transit, reducing colonic residence time and limiting drug exposure at diseased sites.^48^ As a result, CDDS designed under assumptions of normal motility may underperform in clinical disease conditions, highlighting the importance of disease-adaptive formulation design.

Clinical experience has revealed failure modes associated with both pH-responsive and time-dependent delivery systems. Premature drug release in the distal ileum, incomplete colonic targeting, and high inter-patient variability have been reported, particularly in patients with altered gastrointestinal physiology.^49^ These failures emphasize the limitations of single-trigger systems and support the growing shift toward more sophisticated delivery strategies.

To address these challenges, hybrid delivery platforms integrating multiple targeting mechanisms—such as pH sensitivity combined with microbial or enzymatic activation—have gained increasing attention. By responding to more than one physiological cue, hybrid systems offer improved robustness against variability in pH, transit time, and microbiota composition.^50^ Such approaches enhance targeting precision and reduce the likelihood of therapeutic failure.

The formulation strategies included in this review were selected based on mechanistic relevance, translational potential, clinical applicability, and regulatory feasibility, rather than novelty alone.^51^ While classical systems provide foundational insights, emerging technologies such as microbiome-responsive CDDS, hybrid trigger platforms, and AI-assisted formulation design are increasingly recognized as promising directions for next-generation colon-targeted therapies.^52^

Collectively, these considerations highlight that successful CDDS design requires a comprehensive, physiology-driven approach that integrates multiple biological barriers, acknowledges patient variability, and prioritizes translational relevance. Addressing these foundational challenges is essential for advancing colon-specific drug delivery from experimental formulations to clinically reliable therapies.^53^

## 3 Approaches for colon-targeted drug delivery

This section provides an overview of the major formulation strategies developed for colon-targeted drug delivery. These approaches are broadly categorized based on their underlying release mechanisms, including pH responsiveness, time dependency, microbial activation, prodrug strategies, and pressure-controlled systems. The following subsections discuss each approach in detail.

### 3.1 pH-responsive polymer-coated colon drug delivery

**Fig. 2:**
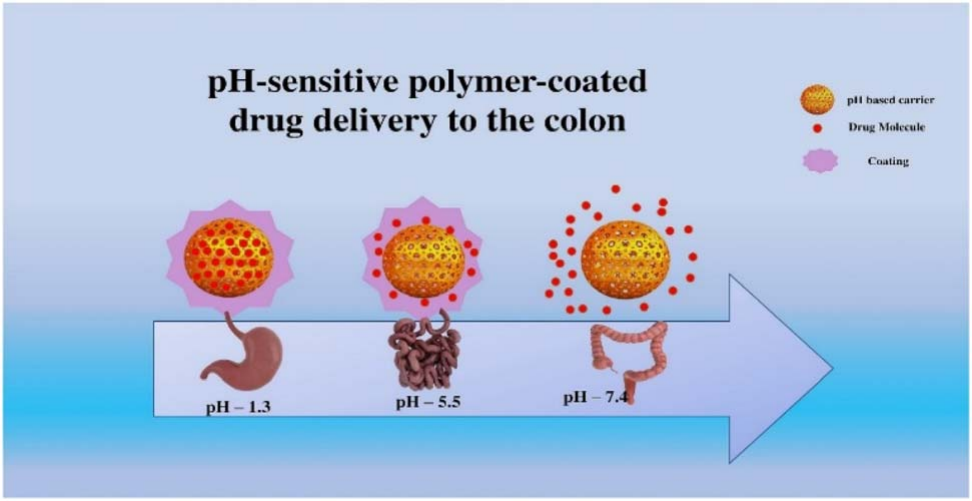
General understanding of pH-sensitive drug delivery approaches for colonic disorders.

The pH-responsive polymer-coated colon drug delivery system provides numerous benefits for colon drug delivery, including targeted drug administration, decreased adverse effects, increased bioavailability, and improved patient compliance. This system is intended to discharge the drug exclusively in the colon, where the pH is lower, thereby minimizing systemic exposure and maximizing local concentration. Additionally, it reduces adverse effects associated with systemic drug delivery, prevents drug degradation in the stomach and small intestine, and improves patient compliance. Overall, the pH-responsive polymer-coated colon drug delivery system offers several benefits that can enhance the efficacy and safety of drug delivery to the colon, making it an attractive treatment option for various colon-related diseases. While fasting, the stomach acidity is between 1 and 2, but it elevates to around 3 after ingesting. Compared to the ileum, the pH of the colon is significantly lower. pH-dependent polymers are employed to respond to these variations.^54^ Polymers used for intestinal targeted delivery are highly insoluble in acidic environments and even more so in basic environments. The solubility of polymers is low in acidic environments but increases as the pH rises. Polymer coatings are unaffected by gastric acid, but they ionize and degrade above a certain pH threshold. The pH at which a polymer dissolves corresponds to the solubility of the polymer. For drug delivery in the ileum and colon, high-pH-tolerance polymers are utilized.^36,55^ Furthermore, Fig. 2 shows how the polymer coat protects the carrier and drug from the external environment; at an appropriate pH, it dissolves and releases the drug.

### 3.2 Time-dependent colon drug delivery system

Time-dependent drug delivery systems (TDDS) are drug delivery systems designed to discharge medications at a predetermined rate over a specific period. Typically, these systems are used to administer medications that must be steadily released over an extended period to accomplish therapeutic effects or to prevent toxicity. Colon-specific drug delivery is one area where time-dependent drug delivery systems have been extensively studied. Improved therapeutic efficacy, decreased dosing frequency, targeted drug delivery, and decreased systemic exposure are advantages of time-dependent drug delivery systems for the colon. Overall, time-dependent drug delivery systems have several advantages for colon-specific drug delivery, and ongoing research in this area holds great promise for developing more effective treatments for colon diseases. Time-controlled release devices are another option for administering medicine.^56,57^ There are constraints on colonic availability when using these methods because of individual differences in stomach emptying time. Inflammatory bowel disease, carcinoid syndrome, diarrhea, and ulcerative colitis are associated with increased colonic motility. Time-based delivery techniques that attempt to alleviate gastrointestinal issues are ineffective. Combining pH-sensitive and time-specific release features in a single dosage form may improve the site specificity of drug administration since the small intestine has a transit period of roughly 31 h and is less variable.^58^ After the stomach is empty, the drug carrier goes to the duodenum, which distributes the medication regularly. A dosage form should regulate medication release in the stomach depending on its capacity to detect acidity to minimize changes in gastric residence duration (acid resistance). An enteric coating protects the medication from stomach acid, preventing its release.^59^

Naeem *et al.* developed and evaluated dual pH/time-dependent nanoparticles (NPs) loaded with budesonide to treat colitis. Eudragit FS30D was employed as a pH-dependent polymer, whereas Eudragit RS100 was employed as a time-dependent controlled-release polymer. In a mouse model of DSS-induced colitis, pH/Time-dependent NPs prevented an abrupt drug release and demonstrated sustained drug release at a colonic pH.^60^ In addition, Sardou *et al.* utilized a 32-factorial design to determine the optimal coating composition and level for colon-specific delivery of 5-aminosalicylic acid (5-ASA) granules. Regarding the colitis activity index and the colon's tissue MDA and GSH enzymes, coated pellets have provided a superior therapeutic outcome than commercially available colonic delivery systems and uncoated pellets. The coating composed of enteric and pH-dependent polymethacrylates could modulate the drug release rate from 5-ASA-coated particles and activate drug release according to pH and time. This indicates that a coating composed of enteric and pH-dependent polymethacrylates could adjust the drug release rate from 5-ASA-coated granules and initiate drug release based on pH and time.^61^ Akhgari *et al.* also evaluated the combination of pH-dependent and time-dependent polymers as a singular coating for developing an indomethacin particle delivery system for the colon. Eudragit S100 and Eudragit L100 were employed as pH-dependent polymers, whereas Eudragit RS was utilized as a time-dependent polymer. They were optimizing formulations utilizing a statistically complete factorial design. The addition of Eudragit RS to pH-dependent polymers allowed for drug release regulation in the colon, as shown by dissolution studies. The optimal formulation was 20% Eudragit RS, 64% Eudragit S, and 16% Eudragit L at a coating level of 10%. Experiments *in vitro* demonstrated that the proposed coating of polymethacrylate polymer dependent on both time and pH may serve as a colonic delivery system for indomethacin.^62^

### 3.3 Microbially triggered drug delivery system

Microbially triggered colon-targeted drug delivery systems are designed to discharge drugs in the colon when specific bacteria are present. This strategy has received considerable attention to enhance the efficacy of medications used to treat diseases that predominantly impact the colon, such as inflammatory bowel disease and colon cancer. The concept behind microbially triggered drug delivery is to initiate the discharge of medications from a targeted delivery system using bacteria that are naturally present in the colon. These systems can discharge medicines over an extended period, increasing patient compliance and decreasing the need for frequent dosage.^63^ Overall, microbially triggered colon-targeted drug delivery systems have the potential to revolutionize the treatment of diseases of the colon by enhancing the efficacy and safety of drug therapies. In terms of their ability to effectively target and treat diseases of the colon, colon-targeted drug delivery systems triggered by microbes have several advantages over conventional drug delivery methods. These include enhanced drug targeting, decreased drug degradation, regulated drug release, and enhanced patient outcomes. These systems can be designed to protect medications from degradation, increase patient adherence, and reduce healthcare costs. However, additional research is required to optimize these delivery systems and assure their human safety and efficacy.^64^

Corrie *et al.* developed an orally administered solid self-nano emulsifying drug delivery system (S-SNEDDS) containing curcumin (CCM) that targets the colon. The size of reconstituted S-SNEDDS droplets was 78.46 nm. Dissolution assays conducted in a medium containing rat caecal contents confirmed the site-specific release of S-SNEDDS powder. A significant cytotoxic effect was observed when CCM-loaded S-SNEDDS was compared to control formulations. A 16s metagenomic technique revealed that fecal microbiota composition did not change substantially before and after lyophilization. The investigation suggested a novel oral formulation based on SNEDDS targeting the colon.^65^ Sinha *et al.*^66^ formulated Matrix tablets containing varying proportions of xanthan gum (XG) and guar gum (GG) and assessed their suitability for colon-specific drug delivery.

As a model, indomethacin was used to evaluate the tablets' ability to inhibit drug release in the upper GIT and undergo enzymatic hydrolysis by colonic flora. The presence of XG in the tablets not only slowed the initial drug release in the upper GIT but also increased susceptibility. There is a growing need to evaluate the efficacy of several delivery methods for protein and peptide-based drugs. Scientists are investigating how these chemicals go through the digestive tract. Pharmacological medication targeted at the colon speeds up the delivery of therapeutic molecules to the organ. Several forms of site-specific medication delivery use local characteristics to trigger drug release at the intended location. Almost 400 distinct types of gut bacteria perform specialized roles in digestion. Researchers are looking toward colon-specific drug delivery systems, using microbial activation as a possible method.^67^ Medication administration to the gut microflora involves a broad range of anaerobic bacteria, including but not limited to *Bacteroides*, *Bifidobacteria*, *Eubacteria*, *Clostridia*, *Enterococci*, *Enterobacteriaceae*, and *Ruminococcin*. Biodegradable polymers are a significant advancement over the prior method of colon-specific medication delivery. Researchers are examining the colon's potential to transfer these chemicals. Many distinct approaches to the delivery of drugs produced by microorganisms are under investigation.^68^

### 3.4 Prodrug strategy for colon drug delivery

A medicine derivative is only as good as the physiological alterations in the human body. The active ingredient in a medication is released from its prodrug after it has been digested and absorbed by the body. It has been theorized that azo compounds are quickly degraded by intestinal flora. Bacteria may degrade gluconolactone, cellulose, and other hydrophobic components, thus linking the medicine to these materials. The creation of prodrugs is dependent on the drug molecules' functional groups. Prodrug carriers need to be tested *in vivo* for drug activation by enzymes and other mechanisms.^69,70^ The hydrolysis of drugs in the colon frees them for intestinal absorption. In 1942, sulfasalazine was shown to be beneficial for those with inflammatory bowel disease. Sulfasalazine is an anti-inflammatory drug that uses sulphapyridine (SP) as a vehicle for the active ingredient, 5-aminosalicylic acid (5-ASA) (5-ASA and SP are bacterial breakdown products of sulphasalazine). The colon connects different azos in the body. The hydrolysis of azo linkages forms prodrugs. The NH and COOH were attached to colon-targeted drug delivery vehicles.

Bacteria in the intestines release the medicine from binding to amino acids.^71,72^ Glycosides are glucose, galactose, and cellobiose. The colon is the glycosides' principal target. In the cecum and colon, bacteria destroy drug–sugar glycosyl linkages.^73^ Recent research has focused on polymers as drug transporters in the colon. Natural and artificial polymers are both used. Polymeric prodrugs have been created using semisynthetic polymers. Compliance with the CDDS was assessed. Numerous azo polymers have been investigated for use as inner coatings for pharmaceuticals. They are susceptible to the actions of the enzyme azo-reductases in the large intestine.^74^

The stomach acid and intestinal enzymes cannot break down peptide capsules coated with azo-aromatic polymers. The chemical is released in the colon when azo bonds are cleaved. The colonic flora's glycosidase activity and steroid glycosides facilitate the effective delivery of medicines to the colon.^75^ Hydrophilic pharmacological glycoside absorption in the intestines is poor. The mucosa may be able to absorb a drug if it reaches the colon and is then broken down by bacterial glycosidases. Various azo polymers have been investigated for potential use as coatings for intramuscularly administered medications. In the large intestine, they are vulnerable to the effects of azo-reductase.^76,77^ Peptide capsules coated with azo-aromatic polymers resist stomach and small intestine digestion. The chemical is released when azo links in the colon are broken.^78,79^

Drugs are distributed more evenly throughout the colon because of steroid glycosides and the glycosidase activity of colonic flora. Hydrophilic pharmacological glycosides are poorly absorbed in the small intestine. If a drug makes it to the colon, where bacterial glycosidases might break it down, the mucosa may absorb some of it. People have been shown to express the enzymes d-galactosidase, d-glucosidase, l-arabinofuranosidase, and d-xylopyranosidase. These glycosides are too large and hydrophilic to squeeze past the cell membrane's lipid bilayer. Induction necessitates glucuronide and sulfate conjugation.^80,81^ Lower GIT bacteria produce glucuronidase, the enzyme responsible for glucuronidase medications in the colon.^82^

Various azo polymers have been investigated for potential use as coatings for intramuscularly administered medications. They may be broken down in the large intestine by azo-reductase. The azo-aromatic polymer coating on peptide capsules makes them resistant to digestion in the stomach and the intestines. The chemical is released when azo connections in the colon are severed.^83^ Negligible intestine absorption of hydrophilic pharmacological glycosides is weak. The mucosa may absorb a medicine if it reaches the colon and is destroyed by bacterial glycosidases. Glucuronidation enables the active medication to exit the body and promotes drug re-absorption for colon-targeted delivery.^84,85^ The cyclic oligosaccharide Cyclodextrins (CyDs) bind 1,4-glucosidic links in 6–8 glucose units to improve medicines' solubility, stability, and bioavailability.^86^ Hydrophilic on the inner surface and lipophilic on the outer surface.^87,88^ They generate complexes, including a variety of medicinal medications. After being transformed into saccharides by colonic bacteria, the stomach finally absorbs them. Due to the flexibility and variety of inclusion complexes, children can synthesize them to reduce the negative qualities of medicinal particles. Hydrophilic and ineffective CyDs may act as drug carriers for fast and prolonged release formulations, respectively, whereas hydrophobic CyDs can monitor water-soluble drug release.^89^ The drug courier must be able to get drugs where needed. The development of colon-targeting prodrugs is made possible by using drug-kid conjugates. When given orally, the CyDs-5-ASA conjugate lowered 5-ASA levels in the gastrointestinal tracts of rats relative to when 5-ASA was given orally alone. After avoiding digestion and absorption in the stomach and small intestine, the conjugate underwent site-specific destruction in the cecum and colon. When given orally, 5-ASA and CyD-5-ASA reduced 5-ASA levels in the blood and urine.^90,91^

### 3.5 Pressure controlled drug delivery system (PCDCS)

A drug delivery system that is controlled by pressure and intended for targeting the colon has the potential to provide various benefits based on pharmacological and physiological factors. The benefits encompass heightened drug localization, extended drug release, heightened bioavailability, targeted therapy for colon ailments, and diminished adverse effects compared to systemic drug delivery. Due to the extended transit time of the colon in comparison to the upper gastrointestinal tract, it is feasible to develop a pressure-controlled drug delivery system that can ensure a sustained and controlled release of drugs in the colon for an extended duration.^92^ Furthermore, specific medications demonstrate inadequate assimilation in the proximal digestive system owing to variables such as susceptibility to pH or enzymatic deterioration. Tailored therapy for colon ailments benefits drugs that experience substantial first-pass metabolism or limited solubility in an acidic milieu.^93^ Drug delivery systems controlled by pressure can administer medications to the colon, guaranteeing that therapeutic concentrations are attained at the precise location of the ailment. The adoption of a focused strategy has the potential to enhance effectiveness and mitigate adverse effects on the entire organism. Enhancing patient convenience and adherence can be achieved by reducing the frequency of drug administration. The administration of drugs directly to the colon can serve as a means of reducing systemic exposure and mitigating associated adverse effects. Additional research and empirical investigations are necessary to comprehensively examine and substantiate the prospective advantages of said systems.^26^ Takaya *et al.* developed pressure-controlled colonic administration capsules made of water-insoluble ethyl cellulose. These gadgets work by having an insoluble polymer capsule containing the medicine disintegrate in the gut due to intestinal pressure. The size and density of the capsules have a role in the procedure, as the colon absorbs water more than the small intestine. Medication absorption was delayed by three to five hours when individuals were given pressure-controlled pills.^94–98^

It has also been attempted to hasten drug absorption by applying pressure to the distal gastrointestinal tract. Muscle contractions in the intestinal wall exert pressure for crushing and moving the contents of the intestines,^99–103^. This pressure varies in length and severity throughout the digestive system. The colon has more significant luminal pressure because of the process of making feces.^11,104^ Because of this, techniques have been designed to survive the pressures of the esophagus, stomach, and small intestine, but they fail when exposed to the more significant pressures of the colon. It has been shown that normal, healthy people's intestinal pressure reaches about 110 mm Hg for around 14 s. Ethylcellulose capsules are used for this reason. You may modify the device's resistance and detonation pressure by modifying the capsule's size and the shell's wall thickness. To a lesser extent, dogs and humans have been used in *in vivo* testing. Colon perforation has yet to be established despite the abundance of data that supports the hypothesis.^36^ The systemic availability of medications through colon delivery systems is contingent on drug release from colon delivery systems and drug breakdown in the colonic lumen. The force of fed-state contractions may be sufficient to rupture the stomach capsule.

### 3.6 CODESTM

The CODESTM (Colon-Targeted Drug Delivery System) is an innovative drug delivery system specifically developed to target the colon. The technique presents several benefits grounded in pharmacological and physiological principles, including targeted administration of drugs, regulated drug discharge, and safeguarding drugs against deterioration. The administration of drugs to a specific site of action is of great advantage in the management of certain medical conditions such as ulcerative colitis, Crohn's disease, and colorectal cancer, where targeted therapy is imperative. The implementation of controlled drug release facilitates a sustained and uniform therapeutic outcome, thereby mitigating the necessity for frequent administration of medication. Preserving pharmaceuticals from degradation is crucial, given that the colon exhibits a comparatively neutral pH compared to the stomach and small intestine.^105^ The CODESTM drug delivery system is a theoretical approach that enhances the bioavailability of drugs that experience inadequate absorption in the upper gastrointestinal tract. The process circumvents the obstacles that impede assimilation within the gastric and duodenal regions, thereby enhancing the bioavailability and density of medication in the colonic area.

Additionally, it decreases the drug's systemic exposure, thereby mitigating systemic toxicity and augmenting patient safety and tolerability. Simplifying the dosing regimen and enhancing patient compliance is a notable advantage, particularly for chronic ailments requiring prolonged therapy. It is noteworthy that CODESTM represents a theoretical drug delivery mechanism, and further elucidation or investigation would be necessary to provide precise particulars regarding CODESTM.^37^

The patent-protected CODESTM method of CDDS is developed to solve difficulties in systems sensitive to pH or temporal fluctuations. The CODESTM CDDS formula takes both pH and bacterial presence into account. The CODESTM system is a method for colon-specific drug delivery independent of time and pH. Lactulose is a unique method since it may promote drug release in the colon alone. Often, acid-soluble components and the enteric component Eudragit L encircle the edible core of a tablet. The pill is shielded until the stomach is empty, when the coating quickly dissolves. Beagle dogs were used for the pharmacokinetic analysis of the newly developed CODESTM technology for targeting acetaminophen in the colon. The closest covering to the tablet's core is an acid-soluble polymer (Eudragit E). However, an HPMC barrier layer sits outside the enteric coating. Assuming that lactulose is rapidly converted into short-chain fatty acids after release, this dissolving method is consistent with the *in vitro* predictions. In this study, we looked at what would happen if the Eudragit layer, which lies just below the planet's surface, were to be made thicker. The results corroborate the hypothesis that lowering lactulose production will slow the decomposition of Eudragit coating.^106^

### 3.7 Osmosis-controlled medication delivery to colon (OROS-CT)

Drugs delivered to the colon using the OROS-CT (Alza Corporation) technology can potentially treat or prevent illnesses or achieve hitherto unattainable levels of systemic absorption. Each push–pull OROS-CT device is in a 4 mm stiff gelatin capsule for protection. Separated by a semipermeable membrane, the osmotic push and drug layers comprise each push–pull bilayer unit. Similar to how a drilled hole in the drug layer of the membrane reveals the drug underneath, a drilled adjuvant also exposes the drug beneath. The mechanics of OROS-push-and-pull CT are enclosed in a dissolving gelatin capsule.^107^ The stomach's acidic environment is too severe for the push–pull mechanism, which generally enables efficient water absorption. Water enters the unit, increasing the osmotic push chamber, and the coating dissolves in the small intestine, where the pH is lower (approximately >7). At the same time, a fluid gel forms in the drug compartment.^108^ Osmotic pressure causes the gel to swell, releasing some drugs at a pace proportional to the amount of water passing through the membrane. Each ulcerative colitis push–pull device has a post-gastric delay of three to four hours, which delays drug absorption in the small intestine. After the gadget reaches the colon, the medicine administration procedure may begin. The contents of an OROS-CT device can be released over four hours and distributed throughout the colon. The potential exists for using new phase-transition devices to deliver drugs to the colon.^109^ An osmotic pump with variable porosity and an enteric coating layer of Eudragit L-100 (6%) was developed by Liu *et al.* to prevent stomach acid from breaking down the medication and chitosan with citric acid. MTCT-OP was absorbed at any point in the digestive process due to its semipermeable cellulose acetate membrane around its 4 percent osmotic core. Above a pH of 6, the citric acid in the tablet begins to disintegrate into an acidic aqueous environment as the enteric coating dissolves and the core absorbs water. Medication delivery was thwarted since the chitosan capsules around the membrane perforations did not dissolve. During 12 to 24 hours, the gel was gradually released into the colon as the tablet's core enlarged.^110^

**Fig. 3:**
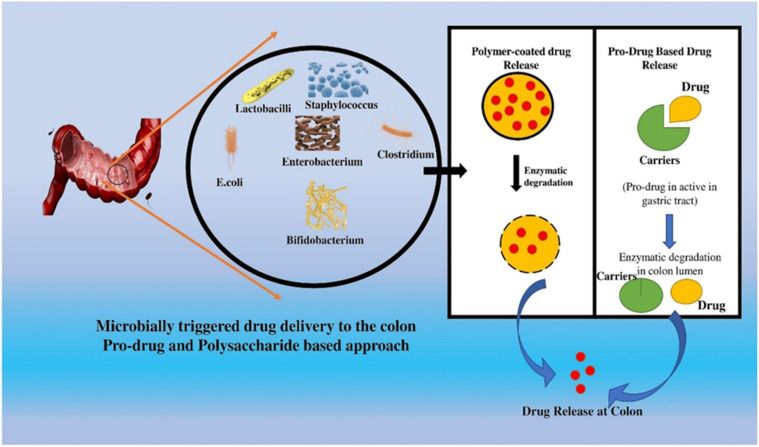
Illustration of microbially initiated medication transport into the colon using the prodrug and polysaccharide strategy.

To evaluate the efficacy of bacteria-activated MTCT-OP, a three-step *in vitro* dissolution experiment was conducted using a basket apparatus that mimics the GIT's physiological circumstances. The drug release rate was inversely related to membrane mass, and the number of holes produced was proportional to the initial concentration of pore-forming material. An enteric-coating membrane protected the formulation's core chitosan until the RCM decomposed it, allowing the cellulose acetate membrane to retain the chitosan. Osmotic pressure was a reliable release monitor, and the MTCT's performance as a drug transporter to the colon was evaluated in circumstances mimicking the gastrointestinal system.^111,112^Fig. 3 further describes the CODETM method of drug administration and the osmotically controlled distribution of drugs into the colon.

### 3.8 Pulsatile drug delivery system

**Fig. 4:**
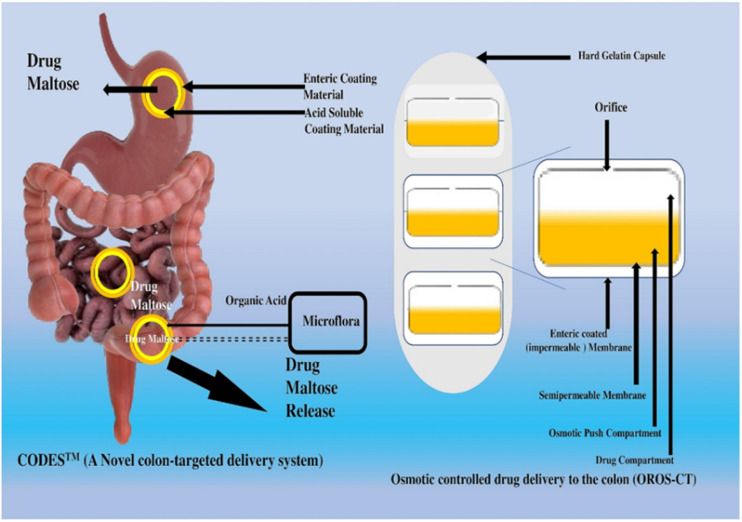
Illustration of the innovative CODESTM delivery system and osmotic-controlled colonic medication delivery.

A drug delivery system controlled by pulsatile release and targeting the colon provides numerous benefits in pharmacology and physiology. The strategies encompass targeted drug administration, timed drug release, improved drug durability, and prolonged drug retention. The colon has been identified as a promising site for drug delivery in specific medical conditions, including IBD, CRC, and various other colonic disorders. Pulsatile-controlled drug delivery systems facilitate accurate and localized administration of drugs to the colon, thereby optimizing therapeutic outcomes and minimizing adverse systemic reactions. The synchronization of drug administration with the body's intrinsic physiological rhythm, known as chronotherapy, can enhance drug effectiveness.^113^ Improved drug stability reduces drug degradation, enhancing bioavailability and therapeutic efficacy. Pulsatile-controlled drug delivery systems have the potential to decrease the frequency of drug administration, mitigate systemic side effects, and enhance patients' quality of life. In addition, they have the potential to assist in the regulation of variations in symptoms, thereby affording individuals enhanced management of their medical condition and augmenting their general state of health. The advantages of a pulsatile controlled drug delivery system for colon targeting are subject to variability based on the system's design, the drug being administered, and the ailment being addressed.^114^ It is imperative to seek the counsel and guidance of a healthcare practitioner or a pharmacist who possesses knowledge of the patient's medical background and requirements to obtain tailored recommendations. Penhasi *et al.* developed a new film coating for pulsatile medication delivery. It comprises water-insoluble hydrophilic particles and a hydrophobic film-forming polymer (FFP). The characteristics of this coating combination were investigated utilizing a sodium diclofenac-containing core as a model drug. The film may rupture after a delay, allowing for a rapid release following the core's dissolution. An enteric coating must be placed over the film coat as an outer layer to guarantee exact site-specific pulsatile dispersion.^76^ To alleviate morning stiffness in rheumatoid arthritis, Aldawsari *et al.* claim to have developed pulsatile ketorolac tromethamine tablets with a compression coating for delayed delivery with an adequate lag time. Compression of polyethylene oxide water-soluble resin (PEO WSR) acted as the coagulant, and Eudragit RLPO compression slowed down the discharge of the final product. *In vitro* X-ray imaging and pharmacokinetic investigations revealed a significant association between coated polymers retaining the right lag time for delayed active release and the suitable lag time required for the dynamic; the optimal compression-coated pulsatile tablet formulation had a nine-hour delay, and 95% of the medication was released 17.42 h after administration.^77^Fig. 4 depicts the PCDCS and pulsatile drug delivery to the colon process.

### 3.9 Nanoparticle-based drug delivery system

Nanoparticle-mediated drug delivery systems designed for colon targeting present numerous benefits from pharmacological and physiological standpoints. The features encompass amplified drug administration, localized effect, heightened drug durability, diminished adverse reactions, extended drug discharge, enhanced drug absorption, and focused treatment. It is possible to design these systems in a manner that facilitates consistent or regulated drug discharge within the colon, thereby decreasing the need for frequent drug dosing and enhancing patient adherence.^78–80^ Furthermore, it is possible to functionalize them with ligands or antibodies that exhibit specific recognition towards receptors or markers that are overexpressed in colon tissues affected by diseases, thereby facilitating targeted therapy. In general, drug delivery systems that utilize nanoparticles for colon targeting present several benefits, including but not limited to improved drug delivery, site-specific action, enhanced drug stability, decreased incidence of side effects, extended drug release, heightened bioavailability, and targeted therapeutic effects.^81^ In ulcerative colitis, nanoparticles are delivered to the inflamed, damaged epithelium of the colon. Reduced efficacy of NPs is caused by their early release, absorption, or degradation in the stomach or intestines. Using a nanoparticles-in-microparticles (NPs in MPs) strategy, Naeem *et al.* created a medication delivery system to treat colitis. Medication release from antibody-coated NPs could be halted in the acidic conditions of the stomach and small intestine.

Nevertheless, they achieved prolonged drug release in a medium with a pH matching that of the ileum and colon. Antibiotic resistance and systemic toxicity complicate attempts to treat inflammatory bowel disease. Nano agents that are non-toxic and simple to administer must be developed to improve IBD treatment.^115^

The CPP's chelation of transition metal ions diminishes the generation of reactive oxygen species, which bodes well for treating inflammatory bowel disease. Covalent assembly of CPP into nanoparticles might enhance CPP's physiology and antioxidative stability (GCPP NPs). A DSS-induced mice colitis model was developed to examine the oral accumulation and therapeutic efficiency of GCPP nips.^83^ Sporopollenin is an underappreciated food-grade polymer. SECs are made by extracting proteins and genetic material from pollen. SECs are empty, hollow particles that may contain small molecule chemicals, proteins, enzymes, and probiotic microorganisms. Covering the particles with lipids, proteins, or polysaccharides may alter the release of SEC, governed by passive diffusion. Ulcerative colitis is characterized by immunological dysregulation in the colon.^116^ According to Wang *et al.*, it is a persistent inflammatory bowel illness of unknown cause. Curcumin (Cur) is a potent anti-inflammatory, but its hydrophobicity and poor absorption prevent it from being widely used. The esterified alginate-curcumin conjugate was made possible by alginate, a naturally occurring water-soluble and biocompatible polymer (Alg-Cur).^117^ Suppressing TLR4 expression in colonic epithelial cells decreased the production and presentation of pro-inflammatory cytokines, reducing inflammation and tissue damage in the colon. Woraphatphadung *et al.* have created two pH-responsive chitosans, *N*-naphthyl-*N*, O-succinyl chitosan (NSCS) and *N*-octyl-*N*, O-succinyl chitosan (NOSC), to inhibit the growth of bacteria by disrupting their cell walls.^118^ (OSCS) Micelles containing polymeric curcumin (CUR) were formed for colonic delivery.

When tested *in vitro* against HT-29 colorectal cancer cells, CUR-modified NSCS exhibited the highest level of anticancer activity. Micelles are pH sensitive, as demonstrated by their form changes in response to different pH-valued solutions. Discharge was dramatically improved by exposure to SIF and SCF (altered colonic fluid). Many obstacles prevent certain cell types from being targeted; however, nano-delivery devices can overcome these problems. It is a promising avenue for overcoming drug resistance in target cells and facilitating the transport of medicines through barriers. However, the difficulty remains in accurately identifying molecular targets and guaranteeing that these chemicals influence just the organs targeted for therapy. In addition, it is essential to understand the drug's fate after being transported to the nucleus and other sensitive cell organelles.

### 3.10 Polysaccharides-based colon targeted drug delivery

**Fig. 5:**
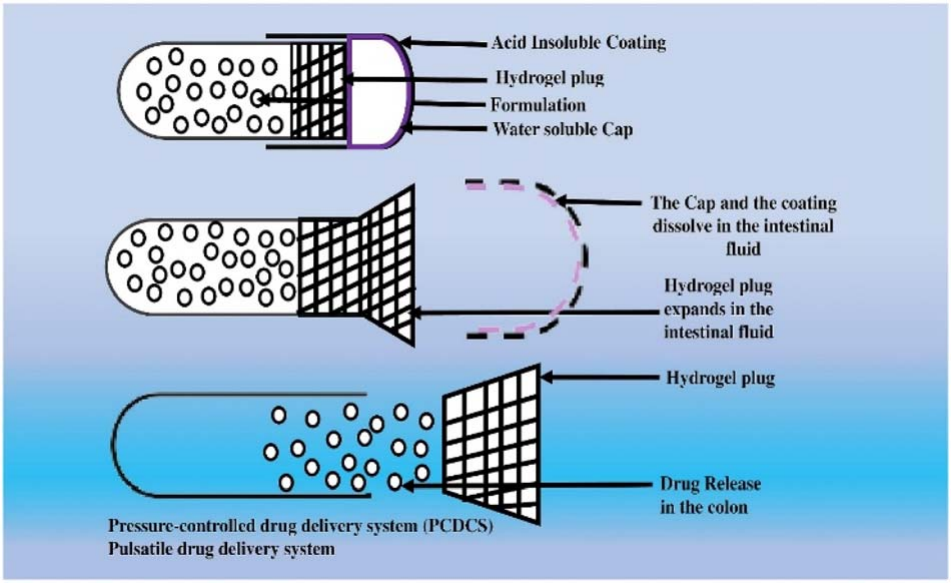
Illustration of the mechanism of action of pressure-controlled drug delivery and pulsatile drug delivery system.

Utilizing natural polysaccharides in colon-targeted drug delivery systems presents various benefits in pharmacology and physiology. The features encompass improved drug targeting, extended drug delivery, safeguarding vulnerable drugs, and targeted management of colon ailments. The mentioned systems can be transformed into diverse drug delivery systems, including hydrogels, microspheres, or coatings, that can offer a continuous or regulated release of drugs. Furthermore, they can safeguard vulnerable pharmaceuticals from deterioration, guaranteeing their unimpaired transportation to the intended efficacy site. Ultimately, they facilitate the administration of pharmaceuticals directly to the impacted region, thereby promoting elevated levels of drug concentration at the intended location and mitigating systemic exposure.^36^ Polysaccharides of natural origin, including but not limited to chitosan, alginate, and pectin, have the potential to function as prebiotics, thereby serving as a source of sustenance for advantageous intestinal microorganisms. The materials are non-toxic, biocompatible, cost-effective, and easily accessible. The benefits have the potential to result in enhanced therapeutic efficacy and diminished systemic adverse reactions. The utilization of natural polysaccharides in colon-targeted drug delivery systems presents several advantages, including improved drug localization, extended drug release, safeguarding of vulnerable drugs, localized treatment of colon-related ailments, interaction with colon microbiota, non-toxicity, biocompatibility, and cost-effectiveness. These advantages make them a promising option for colon-specific drug delivery in diverse pharmacological and physiological scenarios,^119,120^ further depicted in Fig. 5.

There is still significant interest in using these polymers of monosaccharides as medicines since they are numerous, easily accessible, inexpensive, and available in various forms with properties. This includes non-union-toxic polysaccharides (such as guar gum and inulin), animal-based polysaccharides (such as chitosan and chondroitin sulfate), and polysaccharides from algae and bacteria as a colon-targeted therapeutic, naturally occurring polysaccharides have received considerable attention. Due to its hydrophilic, it forms a gel when combined with water and poses no hazard to human health. Simple saccharides are often utilized to target pharmaceuticals to gut bacteria since they are chemically or biochemically stable, harmless, non-toxic, and biodegradable. Algae, microorganisms, chitosan, chondroitin sulfate, guar gum, and inulin are all polysaccharides (dextran).^121^ Sugars like glucose and fructose may be metabolized by bacteria in the gut. Polysaccharide-based strategies protect the drug from the severe upper GIT environment. Hydrolysis of the glycosidic bonds frees the drug moiety from the drug delivery mechanism upon entry into the colon. *Bacteroides* and *Bifidobacterium* are the two most common families of saccharolytic bacteria.

Chitosan, a cationic polymer of poly (*N*-glucosamine) with a very high molecular weight, is extracted from the shells of crabs and shrimp *via* deacetylation. Research into chitosan capsules has been primarily motivated by their potential for use in the intra-colon delivery of medicines.^122^ The specificity of chitosan capsules with an enteric coating has been shown using Wistar rats.^123^ Despite this, several chitosan dosage formulations have been developed for colon-targeted drug delivery.^124^ Mostly made up of (1–4)-linked d-galacturonic acid residues with some 1- and 2-linked l-rhamnose residues thrown in for good measure, pectin is a linear non-starch polysaccharide. Calcium salt decreases pectin's water solubility. Tablets made of a pectin calcium matrix performed well *in vitro*. This matrix design eliminated the need for compressive wrapping of the core.

In contrast, after a pancreatectomy, both insulin tablets rapidly leaked their contents into the dogs' colons.^90^ Giri *et al.* demonstrated that microencapsulated cores composed of biopolymer hydrogel beads, prepared in the presence of calcium ions, enabled controlled release of diclofenac, supporting their potential for colon-specific distribution.^125^ Ulcer development and caecal content were studied *in vitro* using microcapsules of rat caecum containing 0.5% and 2.5% weight-*per*-volume pectin and sodium alginate.^91^ It has been shown that a higher concentration of alginate has a much more profound effect on inhibiting medication release. Six to eight glucose units are linked to Cyclodextrin by (1, 4)-glucosidic bonds. Neither the stomach nor the small intestine can break down or absorb this material. Saccharides are produced from the microflora in the gut by bacteria called *Bacteroides*. The intestinal delivery of these drugs has been studied. For the most part, 1,6-glucopyranosylation occurs along the linear polymer backbone of Dextrans. Dextranases break these glycosidic linkages. These compounds have also been studied as prodrugs for potential new dosage forms. Polysaccharide derived from the galactomannan pathway, with a 2 : 1 ratio of -d-mannopyranose to -d-galactopyranosyl units (thus called “galactomannan”). Possible digestion by bacteria in the large intestine.^126,127^ Formulations of guar gum in microcapsules and matrix tablets have been studied to deliver poorly water-soluble medicines to the colon. Compression coating research shows this polymer is excellent for the uses mentioned earlier. As *Bifidobacteria* make up about 25% of healthy human gut flora, inulin hydrogels have been studied for their possible use as medication transporters in the colon.^128^ The use of azo-crosslinked inulin as a medication carrier in the colon has been investigated recently.^129^ Curcumin has remarkable medicinal potential but needs to be better absorbed since it is insoluble in water. Iurciuc-Tincu *et al.* aimed to increase curcumin's stability and bioavailability by encapsulating it in new polysaccharide-based microparticles. It is possible to provide therapeutic microparticles to the colon by oral ingestion.^130^

### 3.11 Comparison of different approaches for colon-targeted drug delivery

The colon has been identified as a promising site for drug delivery in specific pathological states, including inflammatory bowel disease, colon cancer, and localized infections. Numerous approaches have been devised to attain efficacious drug administration to the colon, including but not limited to coating systems, time-dependent systems, prodrugs, microbial-triggered systems, colon-specific delivery systems, and pressure-controlled colon delivery systems. Coating systems employ variations in pH levels between the gastrointestinal tract and the colon to facilitate drug release, whereas time-dependent systems release the drug after a predetermined time interval. Prodrugs are pharmacologically modified derivatives of drugs that necessitate enzymatic or chemical conversion within the colon to liberate the active form.^131^ Microbial-triggered drug delivery systems employ the existence of enzymes or microbial flora in the colon to initiate the release of drugs. The objective of Colon-Specific Delivery Systems is to achieve targeted drug delivery to the colon by circumventing the release of the drug in the upper gastrointestinal tract.^66^ The Pressure-Controlled Colonic Delivery Systems employ mechanisms dependent on pH and pressure. The technique of magnetic targeting employs a magnetic field to direct the drug toward the colon, thereby facilitating its delivery to the intended site.^132^ Magnetic nanoparticles or carriers with magnetic responsiveness are utilized for drug delivery to the colon.^128,133^ The selection of methodology is contingent upon the medication, intended therapeutic outcome, patient adherence, and underlying ailment under consideration.^134^ The utilization of pH-based systems is prevalent, and their design is relatively uncomplicated, providing regulated drug delivery in reaction to the pH gradient throughout the gastrointestinal tract. Time-based drug delivery systems offer a dependable and anticipated drug release pattern.^135^ However, due to age, diet, and gastrointestinal ailments, the physiological duration of drug transit through the gastrointestinal tract may differ among individuals. Fluctuations in transit duration can potentially impact the temporal release of drugs in time-dependent systems. Collaboration between pharmacologists and physiologists is frequently observed in evaluating various drug delivery methods. Such assessments consider both the pharmacological aspects of drug release and the physiological factors that impact drug behavior within the body.^135^

Microbial-Triggered Systems and Colonic Pressure-Activated Systems are two drug delivery methods that take advantage of the unique conditions present in the colon. The former utilizes the abundance of colonic bacteria to facilitate drug release, while the latter relies on the high pressure in the colon to trigger drug release. Collaboration between pharmacologists and physiologists is standard in evaluating various drug delivery methods. This involves an assessment of the pharmacological properties of drug release and the physiological factors that impact drug behavior within the body. Utilizing natural polysaccharides in drug-delivery systems targeting the colon offers several advantages in the domains of pharmacology and physiology. These benefits include enhanced drug targeting, prolonged drug release, protection of labile drugs, and targeted treatment of colon disorders. The systems possess the potential to be converted into a variety of drug delivery systems, including but not limited to hydrogels, microspheres, or coatings, which can provide a sustained or controlled release of drugs.^135^

Moreover, they can protect susceptible pharmaceuticals from degradation, ensuring their intact conveyance to the designated site of effectiveness. Polysaccharides derived from natural sources, including chitosan, alginate, and pectin, exhibit prebiotic properties and can act as a substrate for intestinal microorganisms to thrive. The application of natural polysaccharides in drug delivery systems targeted towards the colon offers numerous benefits, such as enhanced drug localization, prolonged drug release, protection of labile drugs, localized treatment of colon-specific diseases, interaction with colon microbiota, absence of toxicity, compatibility with biological systems, and economic feasibility. Illustrations depicting various conventional dosage forms, accompanied by their constituent components and specific application areas, are used to evaluate efficacy,^135^ with concise explanations (summarized in Table 1).

**Table 1:** Various conventional dosage forms, their compositions, targeted areas, and evaluation methods, accompanied by concise explanations

Formulation	Composition/drug	Target	Description	Reference
Microporous bilayer osmotic tablet	Dicyclomine hydrochloride and diclofenac potassium	Treatment of IBS	Without any laser drilling, the microporous bilayer osmotic tablet showed acid-resistant, timed release and delivered a drug at an approximate zero order for up to 24 hours	136
Dual release tablet	5-Aminosalicylic acid (5-ASA), caffeine	IBD	CCR tablets with 5-ASA, typically employed in the treatment of IBD, and with the model drug caffeine were evaluated. Whereas 5-ASA absorption reportedly decreases from proximal to distal parts of the intestine, caffeine is absorbed throughout the gastrointestinal tract and is a comparator	137
Zero order sustained release tablets	Budesonide	Colonic IBD	Targeted delivery of budesonide as sustained-release tablets was achieved by coating the tablets with the ColoPulse technology, and results showed effective drug delivery through the entire ileo colon	138
Coated tablets	Infliximab	Ileo-colonic region in IBD	Infliximab containing ColoPulse tablet was satisfactorily efficacious for ileo colonic targeted delivery	139
Matrix tablet	Mesalamine	UC, CD, colorectal cancer	The phosphorylated mandua starch-based matrix tablet prepared by the wet granulation method successfully delivers mesalamine to the colon. The modified starch ensured its targeted delivery to the colon and was identified as a novel drug delivery system targeting the colon	140
Chewable tablets	Bumadizone calcium	UC	The colon-targeted chewable tablet composed of a drug, Eudragit^®^ S100, mannitol, and 50 mg maize starch effectively treated acetic acid-induced ulcerative colitis	141
Multilayer tablet	Paracetamol and 5-ASA	Control of the progression of colitis and alteration of the microbiota	The multilayer oral drug delivery system with Eudragit S outer coating, HPMC inner coating, chitosan, and pectin as microbially degradable components on paracetamol and 5-ASA mini tablets was compelling enough for target delivery of drug to colonic sites	142
Fast disintegrating tablet	Indomethacin	IBD	The formulated FTD containing IND encapsulated within poly(glycerol-adipate-*co*-ω-pentadecalactone), PGA-*co*-PDL, coated with Eudragit L100-55 using o/w single emulsion solvent evaporation technique and coating *via* spray drying indicated the possibility of targeting anti-inflammatory drugs to the colon using FDTs containing microparticles coated with Eudragit	143
Starch-based hydrogel	Ketoprofen	Pain and inflammation at the colonic site	The starch/methacrylic acid (MAAc) copolymer hydrogels were synthesized using γ-rays induced polymerization and crosslinking techniques	144
Konjac gum–xanthan gum–glycerol–sodium alginate hydrogel	Hydrocortisone sodium succinate	Ulcerative colitis	The pH-sensitive hydrogels were shown to release the drug at comparatively higher pH levels, and hydrogels with glycerol showed good colon-targeting activity	145
Carboxymethyl sago pulp pectin hydrogel	Diclofenac sodium	Colon targeting	The hydrogel beads were formed by calcium crosslinking and electron irradiation methods and showed pH-sensitive drug release	146
PVA-based crosslinked hydrogels	Diclofenac sodium, propranolol hydrochloride, and vitamin B6 hydrochloride	Colon targeting	PVA was crosslinked with succinyl chloride, adipoyl, or sebanoyl chloride to form hydrogels that released the drug at pH 2.0, 5.5, and 7.4 by *in vitro* studies	147
Dextran hydrogels	4-Aminobutyric acid, 1,10-diaminodecane	Colon targeting	Cross-linked hydrogels were studied for swelling reversibility within the pH range of 2.0 to 7.4, and the cross-linking efficiencies were between 52–63%	148
Polysaccharide alginate-based hydrogels	Alginate, calcium ions, chondroitin sulfate	Colon targeting	Alginate was cross-linked with Ca^2+^ and chondroitin sulfate to produce alginate-Ca^2+^/CS hydrogels that showed pH-dependent behavior when evaluated on swelling data and morphological analysis	149
Pectin hydrogels beads	Modified pectin, CaCl_2,_ indomethacin	Colon targeting	The hydrogels prepared from commercial citrus and charge-modified pectin showed better oral administration efficiency for drug encapsulation in colon-targeted drug delivery systems	150
Chitosan capsules	5-Amino salicylic acid	Colon targeting	The 5-amino salicylic acid-embedded chitosan capsules were studied to enhance the localization of the drug in the large intestine for healing TNBC-induced colitis in rats. The results showed a satisfactory therapeutic effect	151
Enteric capsules	Oseltamivir	Colon targeting	The anti-influenza drug was targeted for site-specific absorption in the small intestine and colon. The results ensured the high bioavailability of the drug oseltamivir at the desired site	152
Enteric-coated capsule	Chitosan brilliant blue gel	Colon targeting	The enteric-coated capsules with brilliant blue chitosan beads were characterized by γ-scintigraphy to study the colon-targeted drug delivery	153
Capsule	Theophylline	CSDD	The colon-specific capsule made up of Eudragit® RS PO, Eudragit® S100, guar gum, and HPMC was prepared by a dipping process without coating, and the formulation showed to release a little drug in simulated gastric fluids and simulated small intestinal fluids, while most of the drug was released in simulated colonic fluids from CS capsules	154
Phospholipid drug conjugate	Diclofenac	IBD	The PL drug conjugates with the drug and acts as a novel oral drug-targeting approach in IBD therapy	155

### 3.12 Influence of pathophysiology, release mechanisms, and advanced formulation strategies in CDDS

Pathological alterations in gastrointestinal physiology play a decisive role in determining the performance of colon-targeted drug delivery systems (CDDS). Conditions such as intestinal inflammation, diarrhea, and post-surgical resection are associated with accelerated transit time, altered motility, and disrupted mucus architecture, which significantly reduce colonic residence time and drug exposure at diseased sites.^156,157^ Consequently, CDDS designed under healthy physiological assumptions often demonstrate compromised efficacy in active disease states, underscoring the need for disease-adaptive formulation strategies.

Among the various targeting approaches, microbial-triggered delivery systems exploit the dense and metabolically active colonic microbiota to achieve site-specific drug release. These systems rely on enzymes such as azo-reductase, β-glucosidase, β-xylosidase, and dextranase to cleave azo linkages or polysaccharide backbones incorporated into drug carriers or prodrugs.^158^ Experimental studies using fecal fermentation models, animal caecal contents, and *in vivo* imaging have confirmed preferential activation of these systems in the colon.^159^ However, inter-individual variability in microbial composition and enzyme expression leads to heterogeneous drug release profiles, limiting predictability across patient populations.^160^

Nanoparticle-based CDDS offer enhanced drug protection, controlled release, and improved mucosal interaction; however, their stability during gastrointestinal transit remains a major concern. Exposure to acidic pH, bile salts, digestive enzymes, and shear forces may result in premature degradation, aggregation, or drug leakage before reaching the colon.^161^ In addition, mucus entrapment can hinder epithelial access and reduce therapeutic efficacy. Surface modification strategies such as PEGylation, charge modulation, and nanoparticle-in-microparticle (NP-in-MP) systems have been developed to protect nanocarriers during transit and ensure selective colonic release.^162^

Despite promising therapeutic outcomes, the safety and immunogenicity of nanocarrier-based CDDS require careful evaluation. While many carrier materials demonstrate favorable short-term biocompatibility, long-term safety data remain limited. Chronic administration may lead to nanoparticle accumulation, epithelial translocation, or systemic exposure, and the biological impact of degradation products is not yet fully understood.^163^ Comprehensive long-term toxicity, immunogenicity, and biodistribution studies are therefore essential prior to widespread clinical translation.^164^

Pressure-controlled drug delivery systems represent an alternative approach that exploits elevated luminal pressure in the colon generated by fecal compaction and peristaltic activity. These systems remain intact in the stomach and small intestine and rupture selectively in response to colonic pressure, enabling site-specific drug release independent of pH gradients and gastric emptying variability.^165^ Although this strategy offers improved targeting precision, inter-individual differences in colonic motility and pressure profiles necessitate further *in vivo* validation.^166^

Comparative pharmacokinetic studies consistently demonstrate that CDDS can reduce systemic drug exposure while enhancing local colonic drug concentrations compared with conventional oral formulations. These systems often exhibit delayed *T*_max_, reduced *C*_max_, and prolonged local drug residence, which are particularly advantageous for treating localized colonic disorders.^167^ Reduced systemic exposure further contributes to lower off-target toxicity and improved therapeutic indices.

To overcome the limitations of single-trigger systems, hybrid formulations integrating multiple targeting mechanisms—such as pH sensitivity, enzymatic degradation, microbial activation, and time-controlled release—have gained increasing attention. By responding to more than one physiological cue, hybrid CDDS offer improved robustness under conditions of physiological and pathological variability.^168^ However, increased formulation complexity introduces challenges related to manufacturing, scalability, cost, and regulatory approval, emphasizing the importance of early translational planning.^169^

## 4 Recent advances in colon-targeted drug delivery

Research into NDDS and the targeted administration of pharmaceuticals to the colon is vital for treating colon diseases, such as Ulcerative Colitis, Crohn's disease, and colorectal cancer. Advances in NDDS can circumvent these hurdles to effectively distribute medicine for colon illnesses. Many obstacles must be conquered before a colon-specific medication delivery system can be built. There is a good chance that NDDS will be the “magic bullet” that finally removes all the problems, as mentioned earlier. Progress has been made in developing safe and effective colon-targeted NDDS by adjusting their size, shape, surface ligands, and drug-release capabilities. Treatment is more effective and has fewer side effects when distribution methods are improved.^170^ IBD and cancer are treated with NP technology—concerns over clinical effectiveness and intellectual property limit novel pharmacological research, particularly the study of NPs. By giving medication, models that contain pH, bacteria, and receptors are enhanced. Targeted medicine distribution, improved effectiveness, and fewer adverse effects are the norm. These constraints may be bypassed by adopting nanoparticle technology, allowing for effective GI drug delivery.^171^ Using a new pH-responsive PVA/AG/MgO nanocomposite, Moghadam *et al.* successfully delivered doxorubicin to the oral colon. With a composition of 3 mg PVA, 3 mg AG, and 6 mg MgO, the loading efficiency of the resulting nanoparticles was 82%. These micro-bio composites were unparalleled in their pH-sensitive drug release. A DNA aptamer targeting the MUC-1 receptor in the Apt-RBC-HMOS@DOX system.^172^ Fang *et al.* state that cancer treatment benefits from oral medication delivery devices that allow for regulated drug release. Using a green synthesis methodology based on the sol–gel method, MgFe_2_O_4_ nanoparticles with varying calcination temperatures were synthesized utilizing citric acid as a chelating and combustion agent. Hydrogel beads made from a pH-sensitive mixture of carboxymethyl starch and alginate were developed as a potential drug carrier for doxorubicin release in a stomach and small intestine model. An external magnetic field (EMF) might be applied to hasten the drug's exit from the beads. A growing movement is toward using environmentally benign methods to synthesize nanoparticles, avoiding creating and using potentially harmful by-products.^173^ Alwhibi *et al.* explore green synthesis using silver nanoparticles produced from *Commiphora myrrha* (AgNPs). This research found that 30% as much AgNPs in a 100-liter medium for colon cancer therapy produced significantly better outcomes than 100% in the control group.^174^

Khoshtabiat *et al.* created pH-, redox-, and magnetically-responsive nanoplatforms by mixing the chemotherapeutic drugs cisplatin (CDDP) and methotrexate with the Fenton reagent (magnetic hydroxyapatite) (MTX).^175^ For drug administration in the colon, pH-sensitive biopolymers carboxymethyl cellulose (CMC) and chitosan were fashioned into environmentally friendly vehicles (CS). Zinc oxide (ZnO) nanoparticles were encased in CMC beads and coated with a CS layer to create self-assembled core–shell polyelectrolyte complexes. Cancer drug 5-fluorouracil (5-FU) was encapsulated in ZnO, CMC, and CS bio-nanocomposite beads and analyzed using FTIR spectroscopy, scanning electron microscopy/transmission electron microscopy, and thermogravimetric analysis. The porosity of the beads reduced, allowing them to store 5-FU and show signs of acting as they would over a more extended period. Based on the results, ZnO/CMC/CS bio-nanocomposite beads may serve as pH-sensitive, biodegradable carriers of 5-FU.^117^ To target delivery of resveratrol to the colon, the particles developed by Andishmand *et al.* have a zeta potential of +25.01 mV, a particle size of 399.18 nm, and a w/v ratio of 10 : 1 : 3. When PEG was added to the mixture, the average size of the NPs decreased to 8344 nm. This nanocomposite comprises encapsulated tamoxifen citrate (TC) nanofibers and polyvinylpyrrolidone (PVP). Colon-specific delayed release properties of the targeted medication need P2 to be stable in a dissolving medium with a pH of 2.0 and release all the loaded TC in less than 5 minutes in a dissolving solution with a pH of 7.4.^176^ At a polymer-to-drug ratio of 10 : 0.5, the best delayed-release profile was achieved. Tort *et al.* reported on electrospun nanofiber membranes with self-inflating effervescence-based effervescence. In this approach, nanofiber membranes based on self-inflating effervescence are a viable technology for medication administration to the upper gastrointestinal system. Sodium bicarbonate reacts with stomach acid to create carbon dioxide gas, which traps gas bubbles inside the nanofiber network. Nanofibers that float reduce drug release bursts while maintaining drug release. The Parkinson's disease (PD) medication pramipexole is a model treatment.^177^ Tabular depictions in Table 2 portray diverse dosage forms, encompassing their constituent compositions, specific areas of focus, and the corresponding animal/model employed to assess efficacy, accompanied by brief elucidations.

**Table 2:** Various dosage forms, their compositions, targeted areas, and the corresponding animal/model used to evaluate efficacy, with concise descriptions

Dosage form	Composition/drug	Target	Description	Animal model	Reference
Modified gelatin capsules (MGCs)	Aceclofenac	Lower GIT delivery	In rats, the study evaluated biopolymer-based microparticles for aceclofenac delivery using modified gelatin capsules (MGCs). Results showed improved encapsulation efficiency, delayed drug release, and higher anti-inflammatory activity than AC.LPO and AC.SO	Wistar rats	178
Biopolymer based microparticle					
Chitosan nanoparticles	Cromolyn	Colorectal cancer	Cromolyn is prepared in chitosan nanoparticle form by ionic gelation technique to increase its bioavailability	Dimethylhydrazine-induced model in Wistar albino rats	179
Heparin loaded liposomes	Heparin	Active ulcerative colitis	Heparin-loaded liposomes prepared using the film hydration were implemented as intrarectal formulations as enemas target the inflammation in ulcerative colitis	DSS-induced colitis mice	180
Surface-functionalized Caelyx® with anti-EpCAM (SYLC3) aptamer	Doxorubicin liposome	Colon carcinoma	The surface-functionalized PEGylated-nano liposomal doxorubicin (DOX) with anti-EpCAM (epithelial cell adhesion molecule) aptamer *via* post-insertion of anti-EpCAM aptamer-conjugated DSPE-mPEG_2000_ into Caelyx® (ED-lip) showed potentially improved the accumulation of tumor by doxorubicin and increases the animal survival	Mice bearing C26 tumors	181
Folic acid conjugate liposome containing 5FU	5FU, DPPC/cholesterol/FA–PEG–DSPE	Colon cancer	The thin film hydration technique prepares the formulation. The folate-liposomal 5FU enhanced apoptosis of HeLa cells through the mitochondrial signaling pathways, evidenced by the collapse of Δ*Ψ*_m_, the release of cytochrome c, and increased activity of caspases 3/7. *In vivo,* results indicated a significant decrease in tumor volume by targeted liposome, a freer drug that decreases its toxic side effects	BALB/c mice	182
Ca-pectinate beads	Resveratrol	Sphingosine kinase 1 for UC	Ulcerative colitis is a chronic inflammatory bowel disorder with a high risk of colon cancer. A colon-specific delivery formula of resveratrol targets sphingosine kinase 1 and apoptotic pathways, controlling pathogenesis and progression. The study found that resveratrol's anti-inflammatory and apoptotic effects were attributed to its inhibitory effect on SphK1	Oxazolone-induced colitis in Wistar albino rats	183
Natural-lipid (NL) nanoparticle drug delivery system	6-Shogaol	Ulcer colitis	The study encapsulates 6-shogaol in natural-lipid nanoparticles for controlled colon release, showing superior anti-inflammatory efficacy in a mouse colitis model. NL-loaded active metabolites M2 and M13 are potent, potentially developing targeted therapeutic approaches	Female C57BL/6J mice	184
Targeted *θ*-shaped capsule	Indomethacin	Lower GI	New *θ*-shaped capsule with pumping function for controlled hydrophobic drug release, maintaining small intestine indomethacin release paradigm	Healthy New Zealand white rabbits	185
		Small intestine			
Capecitabine-loaded carbon nanotubes	Capecitabine, folic acid, chitosan	Colon cancer	The colon-targeted enteric coated capsule filled with carbon nanotube loaded capecitabine with folic acid gives an exclusive release in the colonic region without premature release in the stomach	*In vivo* roentgenographic study on rabbit	186
Oral pectin/oligochitosan microspheres	Quercetin	Chronic inflammatory bowel syndrome	IBD is a chronic disease with no cure. COS-CaP-QT, a colon-targeted QT delivery system, uses pectin/Ca^2+^ microspheres crosslinked by oligochitosan for pH-dependent drug release. *In vivo*, therapeutic results show relief and intestinal barrier integrity	Wistar albino mice	187
Self-nano emulsifying drug delivery system (SNEDDS)	Genkwanin	Anti-colitis-associated colorectal cancer, colonic tumor	GKA-SNEDDS exhibited enhanced oral bioavailability and excellent anti-CAC efficacy	AOM/DSS-induced CAC mice model	188
Polymeric microspheres	5-FU	Colon cancer	The study developed oral site-specific rate-controlled anticancer drug delivery for colon cancer using double emulsion solvent evaporation. The polymeric microsphere with 5-FU showed excellent physicochemical features, enhancing chemotherapeutic efficacy and reducing side effects	Swiss albino mice	189
Curcumin loaded microsphere	Curcumin, polycaprolactone, poly (vinyl alcohol) hydrolyzed, Eudragit^®^ FS 100	Intestinal inflammation and oxidative stress	The microspheres were prepared using an oil-in-water emulsion technique followed by solvent evaporation and found to reduce the inflammation in the colonic targeted area efficiently	Acetic acid-induced UC in rats	190
Orally administered hydrogel–metal–organic framework hybrids	siRNA-loaded MOF encapsulated in the sodium alginate particles	Site of inflammatory colon	The successful prevention of premature drug release from the MOF-siRNA sodium alginate particles was obtained and resisted the destruction of the payload by gastric and intestinal fluids. The encapsulated cores were perfectly sized to penetrate the colon's mucosal layer	DSS-induced mouse colitis model	191
Low methoxy pectin-dopamine conjugate/konjac glucomannan composite hydrogel microspheres	Olsalazine	Ulcer colitis	Ulcerative colitis (UC) is a chronic inflammatory bowel disease causing intestinal dyshomeostasis. Olsalazine-loaded hydrogel microspheres, formulated intrarectally, improve medication retention and therapeutic impact by scavenging ROS and releasing Zn^2+^ and Olsa for antibacterial and anti-inflammatory actions	Dextran sodium sulfate (DSS)-induced mouse UC model	192

### 4.1 Targeting the colon with porous silicon nanoparticles

Using porous silicon nanoparticles to target the colon is a highly encouraging strategy within drug delivery and therapeutics. Porous silicon nanoparticles exhibit biocompatibility, biodegradability, and a substantial drug-loading capacity, rendering them a viable option for targeted delivery of therapeutic agents to anatomical sites, such as the colon. Selectively targeting the colon with porous silicon nanoparticles encompasses several stages, including loading the nanoparticles with the intended therapeutic agent or drug, oral administration, traversing the stomach and reaching the small intestine, and utilizing pH-sensitive coatings to achieve colon-specific targeting. The implementation of this approach has the potential to enhance the effectiveness of therapy while simultaneously reducing the occurrence of adverse effects on the body as a whole.^193,194^ The utilization of porous silicon nanoparticles in targeted drug delivery exhibits significant promise in enhancing the efficacy of therapeutic interventions for colon-related pathologies. It is possible to engineer drug delivery systems that exhibit stability in the stomach and small intestine while undergoing degradation or drug release in response to the acidic conditions of the colon.

Additionally, these entities can undergo functionalization through the attachment of ligands that exhibit the ability to recognize and bind to receptors or markers that are present in colon tissues or cells.^195^ Upon reaching the colon, nanoparticles can release their drug payload *via* either diffusion or targeted stimuli. Furthermore, the utilization of porous silicon nanoparticles is viable for diagnostic applications in diseases related to the colon. The optimization of nanoparticle design, targeting strategies, and drug release mechanisms is imperative for the effective and safe delivery of drugs to the colon, necessitating research and development efforts.^196,197^

Porous silicon nanoparticles loaded with immunoglobulins and polymers that change pH were created by Kumeria *et al.* Coated with Eudragit L100 or S100 polymers, IgA2-loaded particles are encased in gelatin capsules and released in the intestines and colon, respectively. Compared to polymer-coated nanoparticles, capsule-based formulations maintain 45–54% of the released protein's activity *in vitro*. To achieve optimal anti-inflammatory medicine distribution and absorption in colitis tissue, Chen *et al.* coated porous poly (lactic-*co*-glycolic acid) nanoparticles (NPs) with curcumin after altering the NPs' surface with pluronic F127 (PF127) (CUR) (porous PF127-NPs). Nanoparticles with a calibrated CUR release profile were the goal of their development. They do this by being monodisperse in size (all 270 nm in diameter), negatively charged (very little), and with a small hydrodynamic diameter. The results showed that the modified NPs were biocompatible and aided in improved CUR absorption by cells compared to porous CUR-loaded NPs without PF127 modification.^198^ Leonard *et al.* argue that nanoparticles transport anti-inflammatory drugs to injured epithelial cells, whereas micron-sized particles target inflamed intestinal mucosa. Combining microparticles with nanoparticles may improve the delivery of budesonide, a glucocorticosteroid used to treat IBD. Multi-step S1MP and BNP protocols improved BNP accumulation in inflammatory regions and repaired the barrier function of the Caco-2 inflamed monolayer in a 3D *in vitro* model of inflammatory bowel disease.^199^ Nanoparticles of cerium oxide have both therapeutic and lethal effects on cancer cells. Rasouli *et al.* designed a unique collection of CeO_2_NPs with diverse topologies and morphologies. The hollow core and shell of hCeO_2_/SiO_2_ nanostructures were more cytotoxic than other compounds on the HT-29 cell line, but each of the four compounds was much less hazardous to healthy cells (HFFF2) than cancerous cells (HT-29). Mesoporous silica nanoparticles (MSNs) are flexible delivery vehicles with desirable stability, a wide surface area, and simple encapsulation. The primary deficiencies of MNPs are their explosive drug release and low stability. To reduce their toxic effects and increase their strength, surface ligands may be attached to MNPs.^200^

Using porous silicon nanoparticles (PSiNPs) has facilitated progress in drug design and formulations for colon-targeted drug delivery. The nanoparticles exhibit a distinctive architecture characterized by a substantial surface area and pore volume, facilitating effective loading and encapsulation of pharmaceutical agents. The surface of nanoparticles can undergo modification through functionalization, wherein specific ligands or antibodies are utilized to recognize and bind to receptors in the colon. The nanoparticles can be designed to exploit the physiological conditions of the colon to induce drug release. Nanoparticles of polystyrene (PSiNPs) can be equipped with imaging or contrast agents, which facilitates the identification and observation of anomalies or illnesses in the colon. In general, the utilization of porous silicon nanoparticles (PSiNPs) in drug development and formulations to deliver drugs to the colon exhibits significant promise. However, additional investigation and advancement are required to enhance the design, formulation, and clinical implementation of PSiNPs to achieve efficient colon-targeted drug delivery.

### 4.2 Targeting of the colon with polyphenol-based nanoparticles

Polyphenol-derived nanoparticles possessing colonic targeting attributes represent a distinct category of drug delivery systems engineered to facilitate the targeted delivery of therapeutic agents or drugs to the colon.^201,202^ The nanoparticles are prepared utilizing polyphenols, naturally occurring compounds in various fruits, vegetables, and plants renowned for their advantageous health properties. Polyphenol-based nanoparticles offer a beneficial approach for colonic targeting due to their potential to surmount the obstacles associated with traditional drug delivery techniques. The benefits encompassed by this approach comprise precise targeting, heightened drug stability, regulated release, augmented bioavailability, reduced systemic exposure, and biocompatibility. These characteristics render them promising contenders for advancing efficacious and secure treatments for diverse colon-associated ailments.^203,204^

Microgels and nanogels show promise as drug-delivery vehicles owing to their high surface area, size tunability, and chemical diversity, as reported by Sunar *et al.* Microgel and nano gel carriers may have their release kinetics of active drugs altered by adjusting the kind and concentration of crosslinker. The primary stimuli investigated for their involvement in controlling drug release are pH, solvent, electric fields, magnetic fields, ionic strength, and temperature. Current drug delivery research focuses on studying stimuli-responsive biopolymeric microgels/nanogels.^205^ The polyphenolic chemicals quercetin, rutin, and tannic acid will be reviewed in the context of their use as microgel and nanogel particles for the controlled release of active agents and drug carriers. Upon breakdown by pH, temperature, solvent, or enzyme, these substances may produce phenolic compounds having antibacterial, anticancer, antiallergic, and anti-inflammatory properties.^206^ According to Georgios *et al.*, nanoscale medicine formulations, often known as “nanomedicines”, have reached clinical practice after overcoming significant difficulties in developing novel medical treatments. Nanomedicines have reached clinical practice, but improvements in local effectiveness and toxicity are needed.^207,208^ The fourth generation of nanomedicines is based on biocompatible nanocarriers that are precisely targeted and responsive to external stimuli. Chemical modifications may be applied to improve targeting or therapeutic efficacy.^209–212^ Polyphenols are potent antioxidants, but their limited oral bioavailability and poor permeability limit their utility in medicine.^213^ Miao *et al.* developed polymer-lipid hybrid nanoparticles (PLN) to promote intestine stability, retention, and permeability. EA-PLN (C/S) at a low dose of 6 mg kg^−1^ significantly reduced colonic lipid peroxidation in a mouse model of acute colitis. Colon illnesses and gut dysbiosis may be treated using polyphenol-based drugs. They review the properties and benefits of dietary fibers, the essential processes of encapsulation production, and the recent development of colon-targeted polyphenol delivery systems.^214^ Wen *et al.* propose that electrospun nanofilm might transport proteins with a particular function throughout the colon. Using bovine serum albumin (BSA) as a model protein, coaxial electrospinning generated nanofillers with a core–shell configuration. When added to the simulated intestinal fluid, 75% of the encapsulated BSA was released into the liquid without appreciably affecting its secondary structure. Utilizing systemic, local, and chronotherapeutic drugs to treat the colon is helpful. Due to the location of the colon towards the end of the gastrointestinal (GI) tract, drug distribution is complex.^215^ Cyclodextrin drug conjugates produced by CD covalent bonding with the medication may transport medicines to the colon after oral administration. Several polyphenolic compounds, proteins, and peptides containing carboxyl groups are desirable therapeutic targets—protein.^216^

The potential use of polyphenol-based nanoparticles in drug delivery, particularly colon-targeted drug delivery, has recently garnered considerable interest. Nanoparticles can be engineered with diverse characteristics, such as pH-sensitivity, mucoadhesion, targeting ligands, combination therapies, biocompatibility, biodegradability, and encapsulation. Various approaches are utilized to develop drug design and formulations for colon-targeted drug delivery. These include encapsulation, pH-sensitive properties, mucoadhesive properties, targeting ligands, combination therapies, stability and biocompatibility, and biodegradability. Polyphenol-derived nanoparticles exhibit favorable attributes such as robust stability, biocompatibility, and biodegradability, rendering them well-suited for employment in the pharmaceutical industry. Nevertheless, additional investigation is underway to comprehensively comprehend the potential of these nanoparticles and enhance their configuration for clinical application.

### 4.3 Protein or metal polyphenol-based hollow capsules for targeting the colon

Protein or metal polyphenol-based hollow capsules are a drug delivery system developed to achieve targeted drug release in the colon. The capsules are designed to target the colon for drug delivery specifically. Using capsules in colon-targeted drug delivery presents numerous benefits, including targeted localization, safeguarding of active ingredients, minimized adverse effects, and enhanced bioavailability. The capsules safeguard the active pharmaceutical ingredients, hindering their deterioration or untimely discharge in the proximal section of the digestive system. In addition, they mitigate the exposure of drugs to non-targeted areas of the body, thereby decreasing the likelihood of systemic adverse effects. Hollow capsules based on protein or metal polyphenols present numerous benefits in drug delivery targeted towards the colon. These advantages include safeguarding active components, decreased adverse effects, enhanced bioavailability, regulated release, localized therapy, and improved drug stability.^217^ The capsules in question offer a range of benefits, including targeted delivery to the colon, safeguarding of active ingredients, minimized adverse effects, heightened bioavailability, regulated release, localized therapeutic effects, and increased drug stability. The advantages render them a potentially promising strategy for devising efficacious and focused treatments for colon ailments.^218^ Polyphenols serve as therapeutic medications administered to halt the course of illness and as critical components of drug carriers in modern drug delivery systems. Polyphenols are a group of plant-based bioactive chemicals. Researchers have investigated whether they may help with cancer, inflammation, and heart disease. Potential drawbacks and dangers are thoroughly investigated, including the influence on nano-drug delivery systems' physical and chemical properties and the effects on the body's regular physiological functioning.^219,220^ Polyphenols, the secondary metabolites of plants, are essential to plant function and an essential part of the human diet, as stated by Xu *et al.* Polyphenols encourage a wide variety of molecular and surface interactions, both chemical and physical. They might be utilized as modular building blocks to create new materials with novel structures and features. Pectin is a biopolymer that microbes may use to produce colon-specific transporters. Free therapeutic drug molecules may cause damage to healthy cells, provide insufficient drug concentrations, and be useless in sick areas. Calcium carbonate particles are studied due to their physicochemical and toxicological features; they dissolve slowly due to their brittleness, porosity, and sensitivity to pH. Hydrogels made from soft alginate are moisturizing, bio-adhesive, and not allergenic. The hybrid and carrier models are not identical. Both CaCO_3_ and Alg-Hs are safe biopharmaceuticals that may be made at home. The production and implementation of CaCO_3_ carriers are more labor-intensive. The composite materials used to construct AlgHS carriers give them strength and flexibility.^26^ The stability, release rate, surface functionality, *etc.*, of both chemicals may be improved by coating. Synthesis, properties, production, and use are all discussed by Huang and colleagues. Ulcerative colitis (UC) is an inflammatory bowel disease that causes symptoms without a pattern (IBD). By combining the active components of Turkish gall with FeIII, Liu *et al.* create a gall tannin-metal microcapsule (GTA-FeIII MCP) system for treating UC. Reducing levels of IL-6, IL-1, NO, TNF-, and ROS *in vitro* was achieved using GTA-Fe III MCPs. Mice with *in vivo* UC were treated similarly and showed improvement. This study reveals novel insights into the stability of natural phenolic combinations and solution-based metal sources, and it suggests a new method for administering drugs with anti-UC effects.^221^ Polyphenols are small bioactive chemicals found in plants and are abundant in plant bark, root, leaf, and fruit. Antioxidants, anti-inflammatories, and immunomodulators like polyphenols have been widely used to treat various diseases. Novel drug delivery systems are developed by exploiting the structural and chemical properties of natural active compounds. Polyphenols are critical components of many medication delivery systems that treat various diseases and conditions. Due to their anti-inflammatory, antibacterial, and anti-tumor capabilities, polyphenols are prospective medication and nutritional supplement research leads. This is an excellent alternative formulation for colonic medication delivery.^222^ The development of protein-based and metal polyphenol-based hollow capsules has been facilitated by progress in drug design and formulations for colon-targeted drug delivery. Proteinaceous capsules are commonly fabricated employing either natural or synthetic proteins, such as gelatin, that possess desirable properties such as biocompatibility, biodegradability, and accessibility. It is possible to manipulate their composition to confer specific characteristics, such as regulated drug discharge, durability, and resistance to adverse gastrointestinal environments. The self-assembly of metal ions with polyphenols, specifically tannic acid, results in the formation of capsules based on metal polyphenols. These capsules possess distinctive characteristics attributed to the coordination chemistry between the metal ions and polyphenols. The capsules offer regulated drug release, safeguarding against drug degradation, and the capacity to surmount physiological obstacles in the gastrointestinal tract. Additional investigation and progress within this domain are anticipated to enhance the structure and composition of said capsules, ultimately resulting in enhanced curative effects for individuals afflicted with colon-related ailments.

### 4.4 Polyphenolic compounds loaded polymeric nanoparticles for colon targeting

The distinctive characteristics and benefits of polymer polyphenol nanoparticles have garnered considerable interest in drug delivery, particularly in facilitating effective drug delivery to the colon. The benefits of this approach encompass safeguarding and regulated dispensation, improved bioavailability, precision targeting, limited systemic adverse effects, and elevated drug concentrations at the intended site. Nanoparticles serve as protective vehicles for pharmaceutical agents, impeding their degradation or untimely discharge within the rigorous milieu of the gastrointestinal tract. It is possible to improve the bioavailability of certain substances by prolonging their residence time in the colon, leading to enhanced absorption.^223^ The utilization of site-specific targeting holds significant advantages in the management of medical conditions such as Inflammatory Bowel Disease (IBD), colonic cancer, and colonic infections. Using polymer polyphenol nanoparticles for colonic targeting presents numerous benefits in the context of drug delivery to the colon. These advantages include safeguarding and regulating the release of drugs, increasing bioavailability, targeting specific sites, preserving unstable drugs, regulating drug release, reducing systemic toxicity, and enhancing patient comfort. The benefits render them promising contenders for the advancement of potent and proficient treatments for ailments associated with the colon. Polyphenol polymers can be customized to react to stimuli found in the colon, enabling the development of nanoparticles that exhibit controlled drug release upon arrival at the colon. This facilitates the maintenance of therapeutic concentrations, diminishes the need for frequent dosing, and enhances patient adherence.^224^

The electrospun poly (2-hydroxyethyl methacrylate) (pHEMA) fibers were infused with synthetic and natural antioxidants, as reported by Ghitescu *et al.* Specific polyphenols, such as vanillic, gallic, and syringic acids, catechin, and extract of natural spruce bark, have antioxidant properties. Using 2,2-diphenyl-1-picrylhydrazyl (DPPH), we demonstrated that incorporating antioxidants into pHEMA nanofibers did not affect their inherent antioxidant properties. The breakdown of polyphenols in the environment may be significantly reduced by encapsulation. Rhizomes of the plant *Curcuma longa* contain polyphenol curcumin. It has anti-carcinogenic activity against many cancers and is only one of its many pharmacological actions. However, the novel chemical's potential medical applications are limited because of its poor water solubility and low bioavailability. Polymeric nanocapsules have been used to avoid such complications.^225^ Therefore, Slika *et al.* sought to create two new polymeric nanoparticles (NP) systems that encapsulate either curcumin alone (CURN) or curcumin with piperine (CURPN), which works as a glucuronidation inhibitor and enhances the bioavailability of CUR.^226^ Nanoencapsulation of CUR improved its physiochemical characteristics, drug loading, and drug release. According to *in vitro* and *in vivo* tests, CUR NPs have selective and possibly lethal effects on colon cancer cells. Muhammad *et al.* produced nanoparticles of shellac containing cinnamon bark by precipitating xanthan gum with an anti-solvent.

Antioxidant activity and ferric reduction antioxidant capacity were observed in bioparticles enriched with cinnamon extract, with over 90% of cinnamon's polyphenols released by gastric acid and 90% retained after 20 minutes of heat treatment at 90 °C. Studies on quercetin (*Q*) antioxidant activities showed that silk fibroin nanoparticles might absorb *Q* without damaging its structure. Adjustments were made to the *Q*/SFN ratio, adsorption duration, and solvent combination to optimize the loading of *Q* onto SFNs. Extensive research has shown the ability of SFNs to maintain their nanoscale and antioxidant properties throughout *Q* loading, transit, and gastrointestinal delivery.^227^ Polyphenolic nanoparticles for both DNA and RNA synthesis. Tan *et al.* examine the latest developments in nucleic acid-based drug delivery systems and discuss their promise, specialized applications, and possible risks. DNA/RNA nanotechnology, multivalent nucleic acid nanostructures, and nucleic acid aptamers are all examples of all-nucleic acid designs that might achieve antibody-level targeting by inverting the specific biological features of linear or cyclic nucleic acids. Plants contain polyphenols, a bioactive molecule that has been researched for its potential antioxidant, anti-inflammatory, immunoregulatory, neuroprotective, and cardioprotective activities. Polyphenol-based vehicles are facilitated by the chemical diversity of polyphenol compounds, enabling them to bind and interact with various species. Natural polyphenols have recently emerged as crucial actors.^228^ This review focuses on the rational design and application of polyphenol-based coating films, hollow polyphenol capsules, polyphenol-incorporated hydrogels, and polymer-polyphenol-based nanoparticles (NPs) for the treatment of a wide variety of diseases, including colonic diseases, cancer, infection, cardiovascular disease, and neurodegenerative disorders.

Using polymer polyphenol nanoparticles presents a promising avenue for enhancing drug design and formulations, specifically in drug delivery targeted towards the colon. Polyphenol nanoparticles are a polymeric compound that can be synthetic or natural and possess phenolic functional groups. Their biocompatibility, biodegradability, and capacity to encapsulate diverse drugs have rendered them noteworthy in drug delivery. Polymer polyphenol nanoparticles could encapsulate diverse medicines, creating a protective barrier that prevents drug degradation or premature release. This process ultimately enhances the stability of the drug. The targeting of polymer polyphenol nanoparticles can be improved by modifying or functionalizing ligands or specific moieties, thereby increasing their specificity towards the colon. Polyphenol nanoparticles composed of polymers can be a promising vehicle for targeted drug delivery to the colon. This approach offers several advantages, including protection against enzymatic degradation, minimized systemic side effects, and potential synergistic effects. Surface modifications can be employed to accomplish this objective, for instance, by affixing pH-sensitive polymers that react to the acidic pH environment of the colon. Polyphenols have the potential to offer protection to drugs that are encapsulated by hindering or impeding enzymatic degradation. This can result in an extended release of the drug and better therapeutic results. Moreover, the utilization of polymer polyphenol nanoparticles has the potential to mitigate systemic exposure and its corresponding side effects, thereby augmenting the effectiveness of drugs while concurrently diminishing their harmful impact on healthy tissues. Additional investigation and advancement are required to fully actualize the potential of these nanoparticles in the context of drug delivery targeted toward the colon.

### 4.5 Small molecule nanoparticles derived from polyphenol

**Fig. 6:**
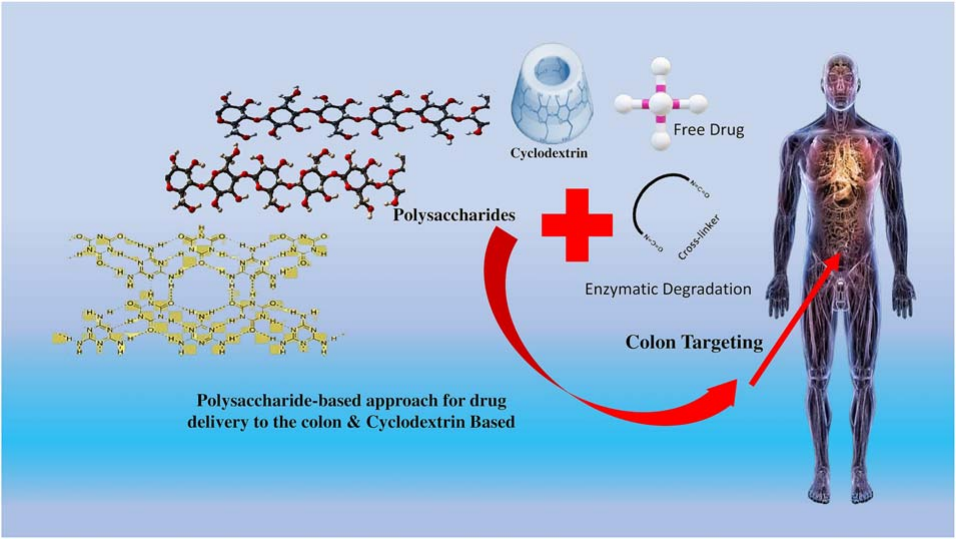
Polysaccharide-based approach with cyclodextrin-based colonic drug delivery.

Polyphenols are a class of organic compounds that occur naturally in various plant-based sources, including fruits, vegetables, and herbs. Their potential advantages, such as the capacity to generate nanoparticles for precise drug delivery to the colon, have rendered them a subject of considerable interest in drug delivery. Polyphenol-derived nanoparticles at the small molecule level present numerous benefits for drug delivery targeting the colon, including improved biocompatibility, release specific to the colon, and heightened drug stability. Polyphenols are a class of naturally occurring compounds widely acknowledged for their biocompatibility and favorable tolerability profile in the human body. Polyphenol nanoparticles can be engineered to achieve colonic-specific release by capitalizing on the physiological attributes of the colon, including but not limited to pH, enzymatic activity, and microbial composition.^220,229^ Polyphenols have been found to exhibit stabilizing properties for drugs, thereby shielding them from degradation or inactivation in the acidic environment of the stomach or the enzymatic activity in the small intestine, thus enhancing drug stability. Polyphenol nanoparticles have emerged as promising contenders for targeted drug delivery to the colon owing to their biocompatibility, colonic-specific release, augmented drug stability, prolonged drug retention, targeted delivery, and potential for combination therapy. Using polyphenol nanoparticles for drug encapsulation can enhance drug stability, prolong drug residence time, decrease targeted delivery, and mitigate systemic side effects. Polyphenol nanoparticles have the potential to function as a platform for combination therapy, wherein a variety of therapeutic agents or drugs can be co-encapsulated and administered to the colon concurrently.^230^ Colon irradiation according to the research by Zhu *et al.*, IBD (inflammatory bowel disease) is increasing at a startling rate throughout the globe. There is currently a lack of effective treatments for IBD, even though it is a significant worldwide health problem. Due to their many useful characteristics, including mass production, good biocompatibility, and pharmacological efficiency, natural active small molecules (NASMs) have been widely employed in treating and preventing IBD. However, they are too hydrophobic, unstable, and have low oral absorption rates. Polymeric nanoparticles (NPs) have recently emerged as a promising substrate for encapsulating NASMs, and a nanocomposite hydrogel composed of graphene oxide and polyvinyl alcohol has been developed to deliver medications to treat colon cancer. Studies of drug release in conditions simulating stomach-to-colon transit revealed that no medicine was released into the gastrointestinal tract before reaching the colon. Hydrogels are a promising new technology that might pave the way for a less intrusive and more effective method of treating colon cancer.^231^Fig. 6 illustrates the different formulations for polyphenol-based colonic medication delivery. Polyphenol-derived nanoparticles at the small molecule level have garnered considerable interest in drug delivery owing to their distinctive characteristics and possible therapeutic uses. Polyphenols are naturally occurring chemical compounds in various plant-based sources, including fruits, vegetables, and tea. These compounds are recognized for their antioxidative and anti-inflammatory characteristics. The development of drug design and formulations for colon-targeted drug delivery has been primarily directed toward enhancing the effectiveness and safety of drug treatments for colon-related ailments, including inflammatory bowel disease, colorectal cancer, and other gastrointestinal disorders. The following are significant developments in drug design and formulations for drug delivery targeted toward the colon. Nanoparticles that are based on polyphenols. Polyphenols exhibit intrinsic properties that render them appropriate for the formulation of nanoparticles. Nanoparticles that are sensitive to pH can be engineered to maintain their stability within the physiological pH of the stomach and small intestine. However, these same nanoparticles can experience disintegration or release of their drug payload when exposed to the mildly acidic environment of the colon. The domain of drug design and formulations for colon-targeted drug delivery is undergoing rapid development, and nanoparticles based on polyphenols exhibit considerable potential for enhancing the efficiency and efficacy of therapeutic interventions for diseases associated with the colon. Nanoparticle systems that exhibit responsiveness to microbial enzymatic activity, mucoadhesive nanoparticles that demonstrate adherence to the mucus layer, and targeted ligands capable of recognizing and binding to receptors that are overexpressed in the colon have been developed by researchers. Using nanoparticles based on polyphenols presents a significant potential for enhancing the efficacy and efficiency of therapeutic interventions targeting colon-related ailments. Further exploration and advancement in this field are anticipated to result in the creation of innovative drug delivery mechanisms that offer improved therapeutic benefits and minimize adverse effects.

### 4.6 Self-micro emulsifying drug delivery system (SMEDDS) for colon targeting

The self-micro emulsifying drug delivery system (SMEDDS) improves inadequately soluble pharmaceutical compounds' solubility, stability, and bioavailability. The term “self-micro emulsifying drug delivery system for colonic targeting (SMEDDS-CT)” refers to a drug delivery system tailored to target the colon specifically. Utilizing a self-micro emulsifying drug delivery system with colon targeting (SMEDDS-CT) offers several benefits, such as heightened drug solubility, improved stability, and targeted delivery. SMEDDS-CT formulations could generate a microemulsion of oil-in-water upon exposure to gastrointestinal fluids, thereby augmenting the dissolution and bioavailability of drugs. Furthermore, SMEDDS-CT formulations provide drug protection against degradation through diverse mechanisms, including the prevention of drug crystallization, the preservation of drug dispersion, and the shielding of the drug from the hostile gastrointestinal milieu.^232^ SMEDDS-CT is a drug delivery system that utilizes self-micro emulsification and is designed for targeted delivery to the colon. This system presents numerous benefits for the delivery of drugs to the colon. The utilization of drug delivery systems has been shown to augment drug solubility, enhance stability, facilitate targeted drug delivery, decrease dosing frequency, permit combination therapy, and offer formulation versatility. The advantages associated with SMEDDS-CT render it a potentially promising strategy for managing colon ailments.^233^ Crohn's disease, IBS, and recurrent *Clostridium difficile* infection are conditions for which fecal microbiota transplantation (FMT) has proven effective. FMT thwarts cancer and inflammation-inducing pathways. Multiple unified formulation approaches have been created to treat colon cancer effectively. Curcumin now has a nanoparticle drug delivery method that is solid and self-emulsifying (S-SNEDDS). Corrie *et al.* created a novel oral formulation with bacteria isolated from human feces and CCM to combat colon cancer.^234^ Curcumin (CUR), although its therapeutic value, is not well absorbed or soluble in water.

To improve CUR's stability, water solubility, and anti-colitis effectiveness, Li *et al.* produced a nano-encapsulated CUR with piperine (PIP) in a SMEDDS. After encasing two hydrophobic components, the CUR-PIP-SMEDDS formulation was analyzed for its color, shape, particle size, zeta potential, and encapsulation efficiency. It was clear that the microemulsion droplets did not merge and remained spherical when treated with CUR-PIP-SMEDDS. CUR and PIP microemulsion droplets generated by SMEDDS had encapsulation efficiencies of 94.34 2.18 and 90.78 2.58%, respectively, with an average droplet size of 15.87 nm. *In vitro*, analysis of CUR-PIP-SMEDDS stability in the colon tissue showed that the combination was more stable when SMEDDS was used as the delivery vehicle and when PIP was co-encapsulated with CUR.^235^

Moreover, a DSS-induced colitis model was used to evaluate the anti-inflammatory effects of CUR-PIP-SMEDDS. CUR-PIP-SMEDDS has been found to prevent colitis when administered to inflammatory colon tissue through retention enema. Based on our findings, creating a colon-specific drug delivery system for CUR could help treat ulcerative colitis. With the potential to increase medication stability and decrease degradation in the stomach environment, SMEDDS may be a practical formulation choice for colon-targeted drug administration. Poorly water-soluble drug types such as curcumin and piperine are encapsulated using the SMEDDS formulation to protect the medicine. Consequently, SMEDDS may be a viable colon-specific delivery method.^236^

The development of innovative strategies for colon-targeted drug delivery, such as ELF (Emulsifying Low-Frequency) micro-emulsifying drug delivery systems, has been facilitated by advancements in drug design and formulations. The systems mentioned above leverage the distinct physiological characteristics of the colon, including its pH, enzymatic activity, and transit time, to achieve accurate drug administration at the intended location. pH-dependent systems employ pH-sensitive polymers that undergo dissolution or swelling in the colon's higher pH environment, releasing the drug at the intended location. Time-dependent drug delivery systems employ various techniques such as compression coating, multiple-unit systems, or osmotic pumps to regulate the release of drugs. The primary focus of this text pertains to the progressions made in drug design and formulations intended for the delivery of drugs to the colon. Notably, utilizing ELF micro-emulsifying drug delivery systems has emerged as a promising approach. The recent advancements in medical technology have created novel opportunities for precise and efficient management of colonic ailments with reduced adverse effects on the body. Drug delivery to the colon can be achieved through various methods, such as microbial-triggered systems, coating techniques, and microemulsions. Various coating techniques, such as enteric and time-dependent coatings, safeguard pharmaceuticals from gastric degradation and facilitate their targeted release in the colon. Microemulsions are dispersions of oil, water, surfactant, and co-surfactant that are thermodynamically stable and isotropic. They provide several benefits, such as improved drug solubility, ease of preparation, and enhanced stability. The recent advancements in medical technology have provided novel opportunities for precise and efficient management of colonic ailments with reduced adverse effects on the body.

### 4.7 Multiparticulate beads as carrier: colon targeting

Multiparticulate beads refer to diminutive, distinct particles or globules that serve as conveyors for drug delivery systems. Using colonic targeting in drug delivery offers numerous benefits, including but not limited to targeted delivery, colonic transit time, localized action, and dose flexibility. Physically, the colon provides a distinctive milieu that multi-particulate bead-based drug delivery systems can effectively leverage. This approach offers a range of advantages, including targeted delivery, localized action, colonic transit time, and dose flexibility. From a pharmacological standpoint, multi-particle beads give the benefit of dose flexibility as they permit accurate regulation of the drug quantity loaded in each bead.^237^ Utilizing multi-particulate beads as carriers for drug delivery with a focus on colonic targeting presents several benefits, including targeted delivery, extended residence time, localized action, dose flexibility, drug compatibility, and reduced variability. The advantages render them a desirable alternative for the creation of productive and user-friendly drug delivery systems that target the colon. Liposomes could entrap various pharmaceutical agents, encompassing hydrophilic and lipophilic compounds, thereby mitigating the likelihood of incomplete drug liberation or erratic absorption.^238,239^

**Fig. 7:**
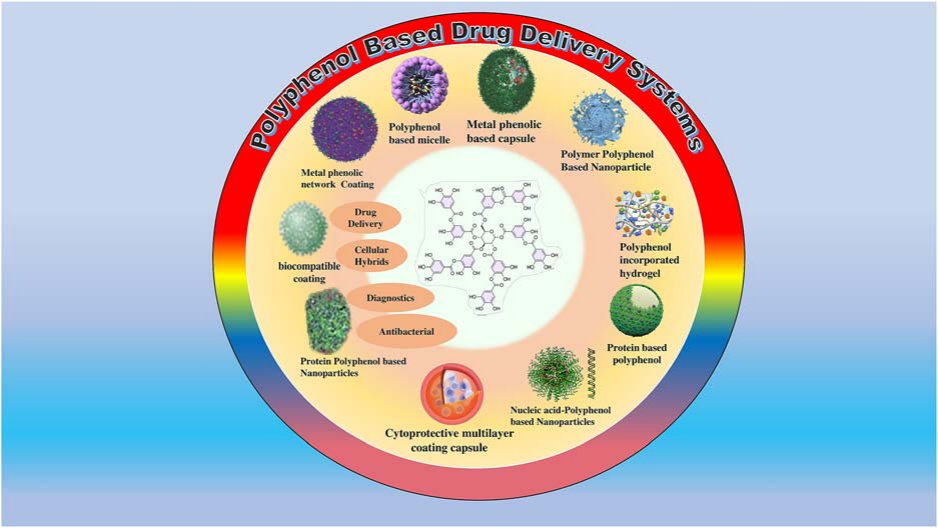
Various polyphenolic based drug delivery targeting colon.

Research into natural polymer-based hydrogels with 3D crosslinked polymeric structures has been extended because of the growing need for biomaterials with specific properties for biomedical applications. Pectin is a biopolymer abundant in the cell walls of fruits and vegetables. It has several applications in the medical, culinary, and textile industries owing to its viscous gel-forming properties. Research into pectin-based biomaterials has been extended because of their potential usefulness in many biomedical contexts, including medicine delivery, wound healing, tissue engineering, implantable devices, and skin-care products. Chitosan is the polymer of choice for injecting active compounds into the colon because of its cationic character, which enhances firm mucosal adhesion. Researchers are exploring therapeutic formulations for the colon to treat inflammatory bowel illness, including chitosan pellets, beads, microspheres, nanoparticles, and drug-polymer conjugates.^240^ Multiparticulate drug delivery systems using chitosan are effective in treating inflammatory bowel disease despite the higher physiological pH found in the colon. It forms multi-unit complexes with gastro-resistance by ionic contact with anionic polymers, allowing for the free transport of payloads to the colon.^241^ Unlike other gastro-resistant polymers on the market, chitosan has been given the GRAS seal of approval, demonstrating its suitability for the long-term management of chronic conditions like IBD. Drug degradation and early drug release before reaching the target location are two methods for restricting the distribution of oral medications to the colon. By combining pH-sensitive beads with nanostructured lipid carriers, Lertpairod *et al.* aimed to create a colon-targeted oral medication delivery system (NLCs). These curcumin-NLCs were encapsulated in pH-sensitive beads using the ionotropic gelation process Eudragit S100. pH-sensitive beads containing NLCs and medicines have been found as a medium for colonic drug delivery.^242^ Preventing treatment from being expelled from the stomach has been found in studies to extend the drug's releasing activity by as much as 12 hours. The drug release kinetics were fitted to a zero-order model based on the Hopfenberg model, demonstrating that the medication was being released at a constant rate owing to polymer breakdown. Pectin generated from callus cultures, as reported by Gunter *et al.*, produces Pectin-Zn Alginate particles with improved gel strength and encapsulating capabilities. Particle growth in simulated stomach fluid is minimal for both GSE-loaded and empty particles (SGF). Fluids showing a healthy colon produced less GSE than those showing colon inflammation (SCF-2.3 and SCF-5.3). Hydrogel devices have shown promise as potential vehicles for colon-specific GSE administration. Chitosan succinate and alginate are studied by Sinha *et al.* for their potential to facilitate the targeted administration of capecitabine to colon cancer. The effects of three parameters, CS (A; X1), CaCl_2_ (B; X2), and SA, on the response variables EE, Size, and Y3, were evaluated using a Box-Behnken design to determine the optimal CS-SA beads. According to the swelling index, the beads showed optimal expansion at a pH of 7.4 and slightly at an acidic pH, indicating that they inhibit drug release at neutral and basic pH levels.^243^ Ansari *et al.* investigate the efficacy of polymeric beads containing 5-fluorouracil encased in Portulaca mucilage for regulating drug release in the colon. Pterostilbene from optimized-coated beads required 14 hours to enter plasma and 22 hours to achieve *C*_max_ (*T*_max_), but the pectin from uncoated beads took just 3 hours. Colon-specific hydrogels may be formed from these naturally occurring polysaccharides, which are biocompatible and biodegradable.^244^Fig. 7 depicts the formulation of multi-particulate beads for colon targeting. In recent years, multi-particle dose administration systems have surpassed single-unit systems in popularity as the preferred method of administering drugs with a specific target organ in mind (the colon). The advantages they are expected to provide are substantial. Benefits include increased bioavailability, decreased potential for systemic toxicity, reduced potential for local irritation, controlled release inside the stomach, and relatively prolonged retention in the upper gastrointestinal tract. Due to the smaller particle size of the active components, these systems exhibit less inter- and intra-subject variability compared to single-unit dosage forms. In addition, the medicine throughout multiparticulate systems is absorbed more uniformly since it is spread more widely in the digestive tract. The fundamental difficulty with the single unit dose form is the unintentional release of the medications in the gastric area due to the various environmental factors in the stomach, intestines, and proximal colon; therefore, the multiarticulate system. The most crucial benefit of multiparticulate medication administration is GI release and absorption uniformity. Thus, colonic targeting is another promising method for medication delivery.

The targeting of the colon has garnered considerable interest in the realm of drug delivery owing to its potential benefits in addressing diverse colonic ailments, including but not limited to IBD, colon cancer, and colonic infections. Multiparticulate beads are constituted by numerous diminutive particles or beads, which have the potential to augment drug release and ameliorate colon-specific targeting. The drug design and formulation field has significantly progressed in developing multi-particulate beads for colon-targeted drug delivery. These advancements include creating pH-dependent, time-controlled, and microbially-triggered systems. The systems mentioned above are engineered to elicit drug release in reaction to pH fluctuations throughout the gastrointestinal tract.

Furthermore, these systems can be tailored to discharge drugs following a predetermined delay period. Multiparticulate beads can be tailored to react to enzymes or bacterial metabolites found in the colon, including but not limited to azo-bonded prodrugs. The utilization of mucoadhesive systems has been shown to effectively extend the retention duration of beads within the colon, thereby augmenting the absorption of drugs. Optimal colon targeting can be achieved through the combination of various approaches. The advancement of multiparticulate bead formulations has been facilitated by introducing novel excipients and technologies, including enteric polymers and innovative manufacturing techniques. Using multiparticulate beads as carriers for colonic targeting in drug delivery presents numerous benefits, such as heightened drug stability, diminished systemic adverse effects, and amplified therapeutic effectiveness. The colon-targeted drug delivery systems field is subject to continuous research and development to explore novel formulations, materials, and techniques to enhance their efficacy.

### 4.8 Liposomes as carrier: colon targeting

The field of pharmaceutical research has witnessed a notable surge in interest in colon-specific drug delivery, owing to its inherent capacity to augment therapeutic efficacy while simultaneously mitigating systemic side effects. Liposomes have emerged as a promising and versatile carrier system for the targeted delivery of drugs to the colon. From the vantage point of a pharmacist, it is of utmost importance to prioritize patient adherence and the preservation of drug safety.^245^ Liposomes facilitate the precise regulation and prolonged dispensation of pharmaceutical agents within the colonic region, conferring notable advantages in managing enduring ailments such as inflammatory bowel disease or colorectal cancer.^246^ Liposomal formulations can shield pharmaceutical compounds from degradation within the highly acidic milieu of the gastric cavity, thereby facilitating a greater proportion of the therapeutic agent to successfully traverse the intricate physiological barriers and ultimately reach its intended destination. By minimizing systemic exposure, liposomes can mitigate adverse effects, augment patient comfort, and promote treatment regimen adherence.^247^ Scientists can meticulously adjust liposomal formulations to attain targeted drug release in the colon by modifying their size, surface charge, and composition. The capacity to functionalize the surfaces of liposomes through the incorporation of ligands or antibodies enables the implementation of active targeting strategies, thereby augmenting the efficacy of drug delivery mechanisms.^248^ Liposomes exhibit immense potential in drug delivery specifically tailored for the colon, presenting advantageous outcomes from the vantage points of pharmacists, researchers, and pharmacologists alike. The capacity to augment drug bioavailability, regulate drug release, and mitigate systemic adverse effects renders them a valuable instrument in the pursuit of enhancing therapies for ailments about the colon. Okamoto *et al.* claim BSA-liposomes were developed as hydrophobic carriers.^249^ The BSA liposomes passively targeted the inflammatory lesions in the colon of DSS-induced colitis model mice during *in vitro* biodistribution tests. In patients with dextran sulfate sodium-induced colitis, intravenous administration of BSA was beneficial.^250^ Oxaliplatin resistance is a significant roadblock in the treatment of colorectal cancer with chemotherapy (CRC). To create a method for the simultaneous delivery of OHP and curcumin, Tiwari *et al.* proposed creating Eudragit S-100 (ES-100) coated alginate beads with drug-loaded targeted liposomes. Liposomal CD44 receptors on CRC cells were targeted by attaching hyaluronic acid to their surface through carbodiimide chemistry.^251^ The MTT assay showed that OC-L-HA was 2.76 times more cytotoxic, and OHP liposomes were 2.58 times more cytotoxic than the targeted CUR.^252^ According to Hodaei *et al.*,^253^ oxaliplatin (OXP) demonstrates significant anticancer activity against colorectal cancer cells. Co-delivery of OXP and HSP to HT-29 colon cancer cells using ochromogum-coated nanoliposomes (COG) enhanced *in vitro* cytotoxic efficacy, indicating improved cellular uptake and therapeutic potential. The zeta potential values of COG formulations prepared at different polymer concentrations suggested acceptable colloidal stability, while nuclear magnetic resonance analysis confirmed successful polymer coating. The coated liposomes remained physically stable for up to 30 days. High encapsulation efficiencies were achieved, with approximately 98% for HSP and 66% for OXP within 24 hours. Bioinformatics analysis using STITCH suggested potential involvement of catalase (CAT)-associated pathways, indicating that assessment of CAT activity may provide insights into the oxidative stress-related mechanisms underlying the observed anticancer effects.^120^ Like Ma *et al.*, they mixed indomethacin, 5-fluorouracil, and curcumin in chitosan/HPMC microcapsules loaded with Eudragit RS NPs. After 6 hours, MCs discharged 10% of their drug-loaded nanoparticles into the simulated intestinal fluid (SIF) and 20% into the simulated colon fluid (SCF) (SCF). In SGF, SIF, and SCF, little curcumin was released from NPs or NP-loaded MCs. MCs show promise as a colon-specific delivery platform for nanomedicines against oral and colorectal cancers.^254^ The encapsulation efficiency of the folic acid-containing liposomes developed by Handali *et al.* was 39.71 percent. Targeted liposomes were shown to limit tumor growth *in vivo* by necrotizing HT-29 cells, which resulted in m-cell collapse, increased cytochrome c activity, and activated caspases. While KGF effectively treated ulcerative colitis, its low drug stability and nonspecific dispersion limited the medicine's usefulness.^255^ Zhao *et al.* encapsulated KGF into liposomes to mimic neutrophils and named their creation KGF-Neus. Encapsulating KGF in KGF Neus resulted in a 95 300.72 percent success rate. KGF neus protein was drawn to the colon of rats by intravenous administration due to dextran sulphate sodium. Gemcitabine and cisplatin are commonly used in combination chemotherapy, although gemcitabine requires intravenous administration. Yang *et al.*^256^ reported a hybrid nanocarrier composed of micelles encapsulated in PEGylated liposomes, demonstrating improved tumor-targeted drug delivery and enhanced anticancer efficacy.^204^

This raises the stakes for developing flexible and tailored nano-DDS for use in precision medicine. Nonetheless, most nanocarriers may be more malleable and selective regarding drug loading. Nanoparticles derived from lipids are preferable because of their particle size, dose, sustained/controlled drug release, and Targeting adaptability. The rapid development of lipid-based nanoparticles for targeted gene therapy, immune therapy, and combination therapies has been made possible by recent advances in genetics, cancer immunology, and cancer pathophysiology. These therapies can potentially eradicate colon cancer and other colon diseases within a short treatment cycle.^257^

The progress in drug design and formulations has resulted in the emergence of diverse, inventive strategies for precise drug delivery, such as using liposomes as conveyors for drug delivery targeted at the colon. Liposomes are a type of spherical vesicle that is comprised of phospholipids. These vesicles can uniquely encapsulate hydrophobic and hydrophilic drugs within their lipid bilayers or aqueous cores. One of the benefits of these materials is their biocompatibility and their versatility in terms of drug encapsulation. Additionally, they have the potential to enhance drug stability and pharmacokinetics. Liposomes can be tailored or engineered to achieve intact delivery to the colon and targeted release of the enclosed drug at the intended site of action in the context of drug delivery targeted to the colon. The aforementioned technological developments encompass pH-responsive liposomes, controlled-release mechanisms based on temporal parameters, and systems that microbial agents activate. The purpose of liposomal formulations is to maintain stability in the upper gastrointestinal tract while undergoing degradation in the presence of colonic bacteria, thereby facilitating drug release. The surface of liposomes can be modified with ligands, including antibodies, peptides, or small molecules, to augment their binding affinity and specificity towards receptors or cells in the colon. The co-encapsulation of excipients can facilitate drug permeation across the colonic mucosa, enhance drug stability, or improve drug absorption. The utilization of liposomes in drug design and formulations has shown potential in improving the management of colon-specific ailments, thereby providing encouraging approaches for colon-targeted drug delivery. However, additional research and development are required to enhance liposomal formulations for clinical application and guarantee their safety and effectiveness.

### 4.9 Hydrogels as the colon-targeted carrier

Hydrogels are polymeric structures composed of hydrophilic polymer chains that absorb and retain substantial quantities of water or biological fluids within a three-dimensional network. Extensive research has been conducted on their properties, and they have been widely employed in diverse biomedical contexts, including drug delivery mechanisms. Hydrogels present various benefits as carriers targeted toward the colon, including but not limited to regulated drug release, drug safeguarding, heightened drug stability, and augmented bioavailability. The attainment of controlled drug release is facilitated through the manipulation of the gel matrix to react to stimuli, whereas safeguarding of drugs is accomplished by the provision of a protective barrier and the augmentation of drug stability. Enhanced bioavailability can be attained through the precise delivery of drugs to the colon.^258^ Hydrogels have emerged as a promising alternative for developing efficient and user-friendly drug delivery systems targeting the colon. The benefits provided by these drug delivery systems include regulated drug release, safeguarding and preservation of drugs, enhanced bioavailability, focused treatment of colonic ailments, decreased frequency of dosing, and mitigated adverse effects. The benefits render hydrogels a compelling alternative for creating productive and user-friendly drug delivery systems targeting the colon. These are especially advantageous for pharmaceuticals that demonstrate inadequate absorption in the upper gastrointestinal tract but possess more excellent absorption rates in the colon.^259^ pH-sensitive pluronic F127 and sodium polystyrene sulfate hydrogels were produced by Suhail *et al.*^260^ for the colonization of 5-aminosalicylic acid. Examining hydrogels' swelling, drug release, and permeability was also a significant focus. The HET-CAM assay was carried out to establish the drug carrier's toxicity. A hydrogel made from hyaluronic acid is discussed for transporting and storing probiotics. Coating *Lactobacillus Reuters*, the prototype probiotic, with hydrogels enhanced its resistance to intestinal conditions. Pectin from yuzu peel was de-esterified using an enzymatic technique, and hydrogel beads were created by ionic gelation with 1, 1.5, and 2% DEYPP. SGF and SIF particles maintained their integrity, but SCF particles disintegrated. Each bead had new crosslinking and polyelectrolyte production; the average particle size and entrapment efficacy increased as de-esterified yuzu peel pectin (DEYPP) concentration rose.^261^ Gautam *et al.* encased calcium carbonate microparticles in a hydrogel to store and release protein drugs in the colon. Pectin/model P, PEG's drug bovine serum albumin (BSA), was first released in the 1980s. Medicines are delivered to the colon using hydrogels manufactured from *Azadirachta* gum; BSA may serve a similar function. Hydrogels from neem gum delay the release of the anti-inflammatory steroid methylprednisolone, reducing side effects and enhancing the colon's inflamed state.^262,263^ Zhao *et al.* created insoluble gel particles that are bioavailable for oral administration. Gel beads made from Eudragit S-100, sodium alginate, and pectin (EU-SA-P) seal the digestive tract and keep digestive juices where they belong. Size, shape, morphology, encapsulation efficiency, swelling, and *in vitro* release were all described in detail. An intestinal fluid-like solution dissolved the beads in three hours, drastically decreasing edema.^264^ To combat hyperthermia and colon cancer *in vivo*, Zheng *et al.* developed an injectable thermosensitive hydrogel. When heated to body temperature, the chitosan (CS) solution injected into the tumor transformed into glycerophosphate (GP). The photothermal and chemotherapeutic characteristics of hydrogels composed of MoS_2_/Bi_2_S_3_-PEG (MBP) and doxorubicin (DOX) nanosheets are well-documented. The resulting composites may be utilized to treat cancer because of the different penetration depths of NIR I and NIR II.^265^ Orally administered medications are discovered to be removed from the body in the feces, as shown by research by Lopez-Molina *et al.* The use of ethylene glycol dimethacrylate (EGDMA) crosslinked guar gum oleate-*graft*-poly (methacrylic acid) resulted in a hydrogel with regulated medicine release that was safe for the colon (GGO-g-PMAc). When placed in a 7.4 pH buffer solution, GGO-pMAc hydrogels released their contents much more quickly than when placed in a 1.2 pH solution. C_3_H_10_T_1/2_ murine mesenchymal stem cells were not harmed by hydrogel concentrations ranging from 0% to 100%.^266^

The distinctive characteristics of hydrogels have rendered them a subject of considerable interest as carriers for drug delivery targeted toward the colon. The hydrogels developed for drug delivery include pH-sensitive hydrogels, biodegradable hydrogels, mucoadhesive hydrogels, microbially triggered hydrogels, multi-layered hydrogels, and targeted drug delivery systems. Hydrogels sensitive to pH have been engineered to maintain their structural integrity within the stomach and small intestine but exhibit swelling and drug discharge within the colon. On the other hand, biodegradable hydrogels have been developed to decompose within the colon and gradually release the drug payload in a regulated manner. The mucoadhesive hydrogels are engineered to exhibit mucoadhesive characteristics, whereas the microbially triggered hydrogels comprise substrates that the colonic bacteria can enzymatically break down. Multi-layered hydrogels offer a means of regulating drug delivery at distinct locations within the gastrointestinal tract. The integration of targeting ligands or antibodies with targeted drug delivery systems can facilitate the precise delivery of drugs to affected regions in the colon. Hydrogels have demonstrated significant promise as carriers for drug delivery explicitly targeted to the colon.

### 4.10 Targeting the colon with microspheres

Pharmaceutical research about colon-specific drug delivery holds great promise in enhancing therapeutic interventions for diseases primarily affecting the colon. Microspheres, alongside various other cutting-edge drug delivery systems, have garnered considerable interest due to their inherent potential in facilitating precise drug targeting to the colon. Microspheres present notable benefits to pharmacists, researchers, and pharmacologists alike, as they effectively shield pharmaceutical compounds from degradation within the highly acidic milieu of the stomach. Additionally, these microspheres enable precise regulation of drug release within the colon, thereby enhancing the bioavailability of drugs at the intended location while concurrently minimizing systemic exposure. These substances have the potential to be formulated into dosage forms that are amenable to patient use, such as capsules, thereby facilitating patient adherence to their prescribed treatment regimens.^267^ Scientists can employ microspheres to encapsulate an extensive array of pharmaceutical substances, encompassing hydrophilic and hydrophobic compounds.

Furthermore, they possess the capacity to customize the dimensions, surface characteristics, and drug release kinetics of these microspheres to cater to diverse therapeutic purposes. The inherent adaptability of this particular attribute facilitates the exploration and implementation of groundbreaking methodologies in pharmaceutical administration, specifically targeting afflictions about the colon, including but not limited to inflammatory bowel disease and colorectal cancer.^268^ Pharmacologists exhibit a keen interest in comprehending the intricate mechanisms of pharmacokinetics and pharmacodynamics about the administration of drugs through microspheres, explicitly targeting the colon. This approach aims to augment drug assimilation and therapeutic effectiveness, thereby mitigating the necessity for frequent administration of doses. Furthermore, it is worth noting that microspheres possess the remarkable capability of accommodating a multitude of pharmaceutical agents, thereby facilitating the implementation of combination therapy strategies to address the intricacies of multifaceted medical conditions. Microspheres possess considerable promise in colon-specific pharmaceutical administration, benefitting the esteemed community of pharmacists, researchers, and pharmacologists alike.^269^

In a study, the intestinal mucosa of rats exposed to ethanol and trinitrobenzene sulfonic acid (TNBS/ethanol) was protected by icariin-containing microspheres. Icariin was released at a rate of 10% in stomach fluid and 65.6% in colonic fluid from the microspheres. Microspheres of crosslinked glutaraldehyde may enhance medication retention in the colon and reduce drug loss in the upper and middle gastrointestinal tracts. As a novel method for preventing and treating ulcerative colitis, this will pave the way for developing similar devices. Yuan *et al.* produced microspheres by infusing CaCl_2_ with konjac glucomannan, sodium alginate, and graphene oxide using high-voltage static electricity. Chitosan increased the tensile strength of microspheres (CS). CaCl_2_ and high-voltage static electricity made KGM, SA, and GO microspheres. Chitosan improved the microspheres' tensile strength (CS) and aided in regulating ciprofloxacin (CPFX) release into fluids, imitating intestinal fluids. According to researchers, a CS coating might prevent the production of folds during lyophilization and increase the substance's stability in SIF and SCF. CS-coated KGM, SA, or GO microspheres may be used for colon-specific drug administration. Due to the solid conjugated interaction between GO and CPFX, adding GO may improve the loading efficiency of spray-dried material.^270^ Alange *et al.* created PAAm-gGK-loaded microspheres for colon-targeted administration of capecitabine. Between 77.3% to 88.74% of the medicines were microencapsulated, and 80% remained in the colon after 24 hours. GK10 microspheres released 19.16% of the treatment five hours after crosslinking with glutaraldehyde (GA); however, only a part of this dose reached the stomach and small intestine; the presence of colonic bacteria on the copolymer increased the excretion of the medication in the rat's feces.^271^ Gadalla *et al.*^272^ suggest encapsulating progesterone in microspheres that target the colon to enhance its oral bioavailability. Entrapment efficiency was 88.8 percent, and the new formulation had a diameter of 1031 meters. The formulation showed pH-dependent swelling, minor drug release in the stomach, and small intestinal fluid models. It thrived on the rat colonic mucosa and rapidly degraded in the rat's caecal contents. Paeoniflorin-encapsulated, Eudragit S100 (EUS100) and halloysite nanotube (HNT)/chitosan (CTS)-coated microspheres may be an efficient method for colon-targeted drug delivery. By utilizing a pH medium and rat colon tissue to mimic stomach and intestinal settings, researchers could evaluate the medication's cumulative release *in vitro*. The HNT networks within the HNT/CTS microspheres were discovered to be three-dimensional.^273^

## 5 Translational, regulatory, and clinical perspectives of colon-targeted drug delivery systems

Despite significant advances in formulation science, the clinical translation of colon-targeted drug delivery systems (CDDS) continues to face substantial challenges. Regulatory approval requires robust *in vitro*–*in vivo* correlation (IVIVC) demonstrating predictable site-specific drug release under physiologically variable conditions, including changes in colonic pH, transit time, and microbiota composition ^274–276^ These challenges are particularly pronounced for nanocarrier-based and hybrid systems, for which regulatory agencies demand extensive toxicological, biodistribution, and long-term safety data prior to clinical approval.^277^

Scalability and manufacturing reproducibility remain critical barriers to commercialization. Advanced CDDS often rely on complex fabrication processes such as multilayer coating, nanoparticle encapsulation, or multi-trigger hybrid architectures, all of which are highly sensitive to process parameters and material variability.^275,278,279^ Batch-to-batch inconsistency can significantly alter release kinetics and compromise product quality. Consequently, early incorporation of Good Manufacturing Practice (GMP) considerations and process analytical technologies is essential to facilitate industrial translation.^280^

From a clinical standpoint, patient compliance and acceptability strongly influence therapeutic outcomes, especially for chronic diseases requiring long-term treatment. Factors such as dosage form size, dosing frequency, and route of administration play decisive roles in adherence.^281^ Oral CDDS are generally favored over rectal formulations, which are often associated with discomfort, inconvenience, and poor long-term compliance despite their localized delivery advantages.^282^

CDDS also offer promising opportunities for the oral delivery of biologics, including peptides and proteins that are otherwise degraded in the upper gastrointestinal tract. By protecting biologics from acidic and enzymatic degradation and enabling release in the relatively milder colonic environment, CDDS may allow non-invasive administration of therapies traditionally limited to injectable routes.^283,284^ The prolonged residence time and reduced protease activity in the colon further support this strategy, although absorption enhancement remains a key challenge.

While inflammatory bowel disease remains the primary therapeutic focus, CDDS have demonstrated potential in colorectal cancer, infectious colitis, and metabolic disorders. Colon-specific delivery enhances local drug concentration at tumor or infection sites while minimizing systemic toxicity, thereby improving therapeutic efficacy.^285^ Emerging nano-based and multifunctional delivery platforms are increasingly being explored for these broader clinical indications.^286^

Environmental and lifestyle factors significantly influence CDDS performance. Diet, concomitant medications (particularly antibiotics and proton pump inhibitors), and lifestyle-related microbiome alterations can modulate colonic pH, enzymatic activity, and transit time, thereby affecting drug release and absorption.^287^ Such variability underscores the importance of adaptive and patient-responsive CDDS designs.

Recent advances in mucoadhesive and mucus-penetrating biomaterials have improved colonic retention and epithelial access. Thiolated polymers, catechol-functionalized polysaccharides, and PEGylated or zwitterionic nanoparticles enhance mucus penetration and prolong residence time at diseased sites.^288^ These properties are particularly beneficial in inflamed colonic tissue, where mucus composition and thickness are altered.

Microbially triggered CDDS face inherent challenges due to inter-individual variability in microbial enzyme expression and regional enzymatic differences along the colon, which can result in inconsistent drug release profiles.^289^ Hybrid systems combining microbial triggers with pH- or time-dependent mechanisms provide improved robustness against such physiological variability.

Nanocarrier-based CDDS, while effective, raise concerns regarding long-term safety and fate. Chronic administration may lead to nanoparticle accumulation, epithelial translocation, or systemic exposure, and the biological impact of degradation products remains insufficiently understood.^290^ Although short-term safety profiles are generally favorable, long-term immunogenicity and bioaccumulation require thorough investigation before widespread clinical adoption.

Effective control of burst release is critical to maintaining colonic targeting specificity and minimizing systemic toxicity. Multilayer coatings, polymer blending, and controlled-release matrices have been successfully employed to regulate diffusion kinetics and ensure gradual drug release in the colon.^291^ Additionally, careful material selection is essential to minimize undesirable immunological reactions, as certain carrier materials may provoke inflammatory responses upon repeated exposure.^292^

Comparative pharmacokinetic studies consistently demonstrate that CDDS can enhance the therapeutic index by maximizing local colonic drug concentrations while reducing systemic exposure.^293^ This pharmacokinetic advantage is particularly valuable for drugs with narrow therapeutic windows or dose-limiting systemic side effects, enabling prolonged therapy and improved clinical outcomes.

Advanced imaging and monitoring techniques, including gamma scintigraphy and magnetic resonance imaging (MRI), are increasingly employed to evaluate *in vivo* CDDS performance. These tools allow non-invasive tracking of dosage form transit, site-specific release, and colonic retention, supporting formulation optimization and IVIVC development.^294^ However, cost and accessibility currently limit their routine clinical application.

Importantly, CDDS performance differs substantially between healthy and diseased colonic environments. Inflammation alters pH, permeability, mucus structure, and motility, which can significantly influence drug release and absorption.^295^ Therefore, disease-relevant evaluation models are essential for ensuring clinical relevance.

Finally, economic feasibility plays a decisive role in clinical adoption. Although advanced CDDS may incur higher manufacturing costs, these can be offset by reduced dosing frequency, fewer adverse effects, lower hospitalization rates, and improved patient quality of life.^296^ Comprehensive cost–benefit analyses are thus critical for supporting clinical implementation.

Overall, future CDDS development should adopt a physiology-driven, patient-centric, and regulatory-aware framework, integrating robust materials, hybrid triggering mechanisms, translational validation, and personalized approaches to overcome current limitations and enable successful clinical translation.^297^

## 6 Challenges and limitations of colon-targeted drug delivery

Colon chyme transit time may be from a few hours to three days in healthy people. Three thick bands comprise the longitudinal layer, stretching from the cecum to the rectosigmoid junction (taeniae). Three taeniae at the rectosigmoid junction surround the rectum. The corrugator cutis ani muscle forms the longitudinal muscle layer in the anal canal. Monkeys, horses, guinea pigs, and rabbits all have taeniae coli, which serve as suspension cables for the muscular arches in their bodies. When the colon contracts, the lumen is reduced by 17%. A circular contraction would decrease the lumen's diameter by a third if the longitudinal muscles were concentric. The coordinated contraction of longitudinal and circular muscles brings on peristalsis. The mesentery supports the colon. A weak mesentery impedes the cecum and colon. Some individuals have a transverse colon that hangs down or a weak sigmoid colon because the mesentery of the transverse and sigmoid colons is more noticeable.^97^

Moreover, concerns about the drug's stability in the intestines may arise. The effectiveness of the therapy may be diminished owing to nonspecific interactions between the drug and the contents of the colon, including meal remnants, digestive juices, mucus, and excrement. The intestinal tract may also contain bacterial enzymes that render the medicine ineffective.^298^

The administration of drugs with a specific target on the colon is a crucial approach in the realm of pharmaceutical advancement, particularly for the management of ailments that primarily affect the colon, such as IBD, colorectal cancer, and irritable bowel syndrome (IBS). The administration of drugs may face several biological and physiological challenges due to the presence of the colon. Overcoming these challenges requires the development of specialized drug delivery modalities such as pH-responsive coatings, prodrugs, bio-adhesive formulations, micro- and nanoparticles, and targeted delivery systems. The efficacy of colon-targeted drug delivery systems can be impacted by various factors, including but not limited to fluctuations in colonic transit time, pH and enzymatic activity, mucus barrier, intestinal motility, and patient compliance. Several factors can impact the efficacy of colon-targeted drug delivery systems, including fluctuations in colonic transit time, pH and enzymatic activity, mucus barrier, intestinal motility, and patient compliance. The achievement of effective commercialization of drug delivery systems that target the colon depends on the ability to surmount various challenges related to regulation and commercialization. The obstacles that need to be overcome in the development and commercialization of medical products encompass a range of issues, such as obtaining regulatory clearance from relevant health agencies, safeguarding intellectual property, expanding production capabilities, guaranteeing market entry and compensation, managing competition, strategizing for the future, performing market research, and executing impactful promotional campaigns. The challenges at hand require interdisciplinary collaboration, rigorous research, and a comprehensive understanding of the specific illness, patient needs, regulatory requirements, and market complexities. Engaging in multidisciplinary cooperation, conducting thorough research, and comprehensively understanding the relevant disease, patient needs, regulatory requirements, and market complexities is essential to address these challenges. Drug delivery systems that target the colon can induce adverse effects such as local irritation, drug toxicity, allergic reactions, systemic absorption, gastrointestinal disturbances, microbial imbalance, drug overdose, and delayed drug absorption. The safety concerns and possible adverse reactions mentioned above highlight the importance of careful formulation design, cautious patient selection, and attentive monitoring when employing colon-specific drug delivery systems. Healthcare professionals must comprehensively assess the benefits and limitations of a specific treatment regimen, considering the distinct medication, route of administration, and patient characteristics, to ensure a safe and effective therapeutic outcome.

## 7 Future perspectives and opportunities in colon-targeted drug delivery

The persistent control of symptoms in the colon, which can result from diseases such as inflammatory bowel disease and colorectal cancer, requires the use of pharmacological interventions that specifically target the colon. This approach can offer advantages concerning patient adherence, safety, and efficacy. Pharmaceutical substances that undergo metabolic processes involving acids and enzymes, like macromolecules, may demonstrate increased effectiveness in their absorption and distribution within the organism. Scholars dedicate considerable effort to investigating systems comprising multiple components and utilizing various approaches to trigger release to address the challenge of pathological variability. The utilization of nanoparticles and microparticles has been observed to augment drug absorption and enhance therapeutic targeting. Using computers or electronic devices to design formulations presents the possibility of cost reduction and time-saving. Undertaking persistent efforts towards developing innovative formulation technologies that can surmount the limitations of existing formulation strategies is of utmost importance. This research investigates diverse formulation approaches for creating colon-targeted delivery systems and presents exemplary implementation cases. Every strategy has its advantages and disadvantages, and each necessitates improvement. Undertaking a comprehensive examination of the physiology and pathophysiology of the colon. In contemporary times, there has been growing scholarly attention towards exploring colon-targeted drug delivery, owing to its capacity to facilitate the management of diverse gastrointestinal disorders and augment the therapeutic potency of pharmaceuticals. Novel drug delivery systems that are specific to the colon, intelligent drug delivery systems, prodrugs that target the colon specifically, and delivery systems that target the microbiota are emerging in the field of drug delivery. The systems can react to diverse stimuli, including but not limited to pH, temperature, enzymes, or biomarkers that are detectable in the colon. This attribute is linked to improved therapeutic results while concurrently reducing adverse effects. Current research endeavors in drug delivery to the colon are primarily directed toward developing sophisticated, customized, and minimally invasive techniques that can accurately administer drugs to the colon, interface with the intestinal microbiota, and enhance treatment efficacy for gastrointestinal pathologies. Current research in drug delivery examines various approaches, such as combination therapies, non-invasive routes of administration, and strategies to improve drug bioavailability and maximize therapeutic effectiveness. A focus on improving the efficacy and safety of drug administration. The text underscores the significance of investigating drug delivery systems and technologies that exhibit potential in precisely targeting the colon.

These systems exhibit the potential to be administered *via* endoscopic or minimally invasive modalities, leading to extended drug exposure to the colon tissue. The application of nano/microcarriers and prodrug-based systems has garnered considerable interest in drug delivery research owing to their capacity to safeguard drugs from degradation and enhance their stability in the gastrointestinal tract. Using colon-specific drug delivery presents numerous prospects and possibilities within pharmaceuticals and healthcare. This includes the treatment of colon-specific pathologies, irritable bowel syndrome, microbial infections, and chronobiological disorders. It is feasible to devise these systems with the capability to dispense drugs at predetermined intervals in synchronization with the circadian rhythm of the colon, safeguard pharmaceuticals from degradation in the stomach and small intestine, enhance pharmacokinetic properties and patient adherence, concentrate drugs for precise effects, and administer biologics and gene therapy. The utilization of drug administration targeted toward the colon offers a diverse array of prospects and possibilities for enhanced and accurate handling of illnesses in the colon, as well as localized afflictions.

## 8 Conclusion and future aspects

Colon-targeted drug delivery systems have emerged as a promising therapeutic approach for various colon-related conditions. These systems provide precise drug administration at the intended site, reduced systemic effects, and improved therapeutic effectiveness. Researchers have made significant progress using various methodologies to achieve efficient colon-targeting, enabling elevated drug concentrations at the desired location while minimizing exposure to non-target tissues. This field falls within Advanced Drug Delivery Systems. Academic researchers can develop innovative techniques that offer enhanced release kinetics, greater resilience, and improved biocompatibility. Combining colon-targeting with other therapeutic modalities can enhance treatment effectiveness. Tailoring delivery systems to individual patient needs aligns with personalized medicine. Diagnostic tools like imaging and molecular profiling can expedite personalized drug delivery approaches. Regulatory considerations are vital to ensure safety and effectiveness. Ethical aspects include patient safety, informed consent, equitable healthcare access, ethical research, and patient-centered care. Researchers must follow ethical guidelines, secure clearances, ensure subject confidentiality, and maintain integrity in publishing. Colon-targeted delivery provides precise drug release in the colon, improving patient outcomes, reducing costs, and benefiting public health. It enhances treatment efficacy for diseases like IBD, IBS, and colorectal cancer, minimizes drug exposure to other organs, reduces side effects, and improves tolerance. Improved drug effectiveness and fewer adverse events lead to better disease management, fewer hospitalizations, and reduced healthcare costs. Nanomedicine research, with ∼1500 patents and clinical trials, focuses on materials with better homogeneity and drug properties. Molecular marker identification and interdisciplinary studies offer promise. Though nanorobots show potential, further research is needed to assess their impacts and costs.

## Declaration

This review article used different language improving software (Grammarly Premium, QuillBot Premium) to improve the grammatical errors.

## Conflicts of interest

The authors declare no conflicts of interest.

## Data Availability

No new data were generated or analysed as part of this review. All data supporting this article are derived from previously published studies, which are cited throughout the manuscript and listed in the references.
